# NSun2‐Mediated tsRNAs Alleviate Liver Fibrosis via FAK Dephosphorylation

**DOI:** 10.1111/cpr.70058

**Published:** 2025-05-12

**Authors:** Pengcheng Li, Sunyang Ying, Yu Zou, Xin Wang, Runxue Zhang, Cheng Huang, Moyu Dai, Kai Xu, Guihai Feng, Xin Li, Haiping Jiang, Zhikun Li, Ying Zhang, Wei Li, Qi Zhou

**Affiliations:** ^1^ College of Life Science Northeast Agricultural University of China Harbin China; ^2^ State key Laboratory of Organ Regeneration and Reconstruction Institute of Zoology, Chinese Academy of Sciences Beijing China; ^3^ University of Chinese Academy of Sciences Beijing China; ^4^ Medical School University of Chinese Academy of Sciences Beijing China; ^5^ Beijing Institute for Stem Cell and Regenerative Medicine Beijing China

**Keywords:** FAK, liver fibrosis, NSun2, sinusoidal capillarization, tsRNA

## Abstract

Sinusoidal capillarization – key symptoms of liver fibrosis progression – represents potential therapeutic targets. tRNA modification‐mediated tRNA‐derived small RNAs (tsRNAs) play a role in angiogenesis. NSun2, an RNA methyltransferase, generates a significant number of tsRNAs. However, the role of NSun2 and its mediated tsRNAs in liver fibrosis remains unclear. In this study, *NSun2* deficiency was found to inhibit sinusoidal capillarization, alleviating liver fibrosis. Furthermore, endothelial cell angiogenesis and migration were disrupted in *NSun2* knockout mice. Mechanistically, reduced *NSun2* expression led to alterations in the functional tsRNAs tRF‐1‐S25 and tRF‐5‐V31, which regulate sinusoidal capillarization by targeting key proteins, including DUSP1 and FAK – crucial clinical targets. Moreover, intravenous injection of tRF‐1‐S25 and tRF‐5‐V31 inhibitor rescued liver fibrosis in mice. In conclusion, tsRNAs generated by NSun2‐mediated modification of tRNAs inhibit sinusoidal capillarization. Furthermore, targeting the DUSP1/FAK/p‐FAK pathway offers an innovative approach to treat this disease.

## Graphical abstract

*NSun2* deficiency inhibited sinusoidal capillarization, alleviating liver fibrosis. Reduced *NSun2* expression led to alterations in the functional tsRNAs tRF‐1‐S25 and tRF‐5‐V31, which regulate sinusoidal capillarization by targeting key proteins, including DUSP1 and FAK – crucial clinical targets – offering a novel RNA‐based therapeutic approach for liver fibrosis.

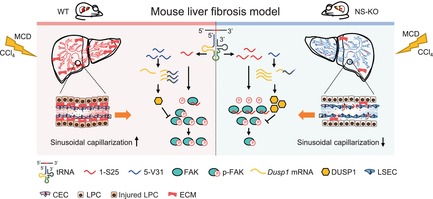

## 1 Introduction

Liver fibrosis, a hallmark of chronic liver injury, represents a critical juncture in the progression towards irreversible liver damage, for example, in the case of liver cirrhosis. This complex condition is typified by excessive deposition of extracellular matrix (ECM) proteins, leading to disruption of normal liver architecture and function [1, 2]. The development of liver fibrosis is closely associated with the activation of hepatic stellate cells (HSCs), which become the principal producers of ECM components [3]. During fibrosis, HSCs are heavily influenced by surrounding structures [4], including the damage and necrosis of adjacent hepatocytes and the recruitment of macrophages, which contribute to HSC activation and increased collagen protein expression [5, 6, 7]. Additionally, sinusoidal capillarization further promotes the secretion of factors that accelerate HSC proliferation and collagen production [8, 9]. Therefore, modulating these structures during injury may serve as a therapeutic strategy for treating liver fibrosis.

The vascular system plays an indispensable role in the treatment of various liver diseases. In liver fibrosis, aberrant angiogenesis leads to sinusoidal capillarization, a process in which fenestrated liver sinusoids become continuous and lose their fenestrations [10]. This change inhibits nutrient transport and exacerbates hepatocyte damage [11]. Simultaneously, liver sinusoidal endothelial cells (LSECs) undergo cytoskeletal reorganisation and acquire migratory capabilities to form continuous structures and transform into capillary endothelial cells (CECs) [12]. These transformed cells secrete numerous factors around HSCs, promoting their activation [13]. This vascular transformation is driven by an imbalance between pro‐angiogenic factors and anti‐angiogenic factors, as well as changes in endogenous factors including mammalian target of rapamycin (mTOR), Src kinase and focal adhesion kinase (FAK) [14, 15]. Notably, some vascular regulatory factors have been utilised in the treatment of diseases. For example, FAK inhibitors have been reported to control cell adhesion and migration, thereby inhibiting aberrant vascular remodelling in tumours and suppressing tumour growth [16]. Hence, targeting the molecular drivers of sinusoidal capillarization presents a promising therapeutic approach to managing liver fibrosis. By modulating angiogenesis, proliferation or migration of endothelial cells, it may be possible to disrupt their pathological interplay in liver fibrosis. However, strategies targeting vascular factors have not yet been applied to the clinical treatment of liver fibrosis.

Recent studies have shown that tRNA‐derived small RNAs (tsRNAs) play crucial roles in cellular processes [17]. These tsRNAs are derived from the precise cleavage of unstable tRNAs that have lost their modifications. For instance, NOP2/Sun RNA methyltransferase 2 (NSun2), a methyltransferase, is vital for the stability and function of tRNAs and can mediate the production of tsRNAs [18]. Specific tRNAs have been shown to interact with the mTOR pathway proteins, which are key regulators of angiogenesis [19]. Furthermore, tsRNAs can influence the expression of key angiogenic factors such as vascular endothelial growth factor (VEGF) and angiopoietins [20, 21]. It is reported that NSun2, through its impact on RNA methylation, also influences angiogenic processes [22]. This implies that tsRNAs activated by the change in *NSun2* downregulation might influence vascular development and remodelling as well as regulate endothelial cell behaviour, consequently impacting crucial steps in sinusoidal capillarization such as endothelial cell tube formation, migration and proliferation.

Given the previously discovered roles of NSun2 and tsRNA in regulating angiogenesis and their impact on fibrosis [23, 24], we reason that tsRNAs whose production is induced by the change in *NSun2* expression might influence the sinusoidal capillarization during liver fibrosis. To prove this hypothesis, in a *NSun2*‐knockout (NS‐KO) mouse model, we screened two tsRNAs that regulate the migration of endothelial cells in order to target the vascular system in the liver. Through the regulation of sinusoidal capillarization, these two tsRNAs alleviate the progression of liver fibrosis by modulating the phosphorylation of the key molecule FAK in angiogenesis. Collectively, the findings untangle the molecular intricacies of tsRNA‐mediated regulation in angiogenesis, exploring their potential as novel biomarkers and therapeutic targets in vascular pathologies during the progression of liver fibrosis.

## 2 Materials and Methods

### 2.1 Animals

B6D2F1/Crl mice were purchased from Beijing Vital River Laboratories and housed at the animal facilities of the Chinese Academy of Sciences. The mice were maintained in a specific pathogen‐free barrier environment with a 12‐h light/12‐h dark cycle, and they had free access to food and water. All experiments used age‐ and weight‐matched male littermate mice. All animal studies were conducted in accordance with the Guidelines for Reporting of In Vivo Experiments (ARRIVE) developed by the National Centre for the Replacement, Refinement and Reduction of Animals in Research (NC3Rs) and the ethical criteria set by the Institutional Animal Care and Use Committee (IACUC) at the Institute of Zoology, Chinese Academy of Sciences.

### 2.2 Mouse Fibrosis Model Construction

Liver fibrosis was induced in mice using two methods: repeated injections of carbon tetrachloride (CCl_4_) and dietary intervention. For CCl_4_‐induced fibrosis, a 40% concentration of CCl_4_ (ACMEC, C27711‐500 mL) was prepared by diluting it with corn oil. Mice were intraperitoneally injected with a dosage of 2.5 mL/kg of the CCl_4_ solution. The control group received injections of corn oil only. Injections were administered twice a week for a total of four consecutive weeks. Mice were sacrificed 2 days after the last injection for further analysis. Additionally, a dietary intervention was applied to induce fibrosis: the experimental group was fed a methionine choline‐deficient (MCD) feed, whilst the control group received normal feed. The feeding regimen was continued for a period of 4 weeks. After 4 weeks of feeding, blood samples were collected; subsequently, the mice were sacrificed for further evaluation.

### 2.3 Immunofluorescence Staining of Paraffin Sections

Fresh tissue specimens were fixed in 4% PFA for more than 24 h, followed by dehydration with graded ethanol and embedding in wax. The wax blocks were then sliced into 4 μm sections using a microtome. Paraffin sections were dewaxed and subjected to high‐temperature and high‐pressure antigen retrieval in sodium citrate buffer (Solarbio, C1032) for 10 min, using a pressure cooker. The sections were outlined with a histochemical pen and incubated in TBS (2% BSA, Sigma, A1933; 0.3% Triton‐X100, Sigma, X100) for 1 h. Subsequently, the sections were washed three times with PBS and incubated overnight at 4°C with a 1:200 dilution of the primary antibodies (CD31, Abcam, ab28364; CD34, Solarbio, K009404P; α‐SMA, Abcam, ab5694; VE‐cad, AF1002, R&D system) in TBS. Next, the sections were washed three times with PBS. The sections were then incubated with the secondary antibody (donkey anti‐rabbit 488, Invitrogen, A21206; donkey anti‐mouse 546, Invitrogen, A10036) at 25°C for 1 h in the dark. Cell nuclei were stained with DAPI for 10 min, followed by a washing step in PBS. To prevent fluorescence fading, the sections were blocked with an anti‐fluorescent quencher (Servicebio, G1401) and stored at −20°C. Images were acquired, examined and analysed using a fluorescence microscope.

### 2.4 Isolation of Mouse Liver Parenchymal Cells and Liver Sinusoidal Endothelial Cells

To isolate mouse liver parenchymal cells (LPCs) and LSECs, mice were subjected to 16–24 h fasting and then anaesthetised. The abdominal cavity was opened, and a venous retention needle filled with Ca^2+^‐free perfusate (Gibco, 14170161), preheated to 37°C, was inserted into the portal vein. Once the liver was confirmed to be full and enlarged, the inferior vena cava was swiftly severed, and the liver was perfused at a flow rate of 2 mL/min. After confirming that the flushed liquid was colourless, the perfusion solution was replaced with a digestion solution containing collagenase IV (Sigma, C5138‐1G). When the liver became soft, it was removed and shredded. The shredded liver was then soaked in 1 mL of digestion solution containing collagenase IV at 37°C for 3 min. Subsequently, 3 mL of mouse liver sinusoidal endothelial medium (Procell, CM‐M040) was added, and the mixture was gently agitated. The resulting solution was sequentially passed through 100 μm and 40 μm cell sieves to obtain a single‐cell suspension. The single‐cell suspension was centrifuged at 50 × *g* for 20 min. The pellet containing LPC was collected and plated in a culture dish pre‐coated with collagen I (Corning, 354236) that had been incubated overnight. Mouse liver parenchymal medium (Procell, CM‐M033) was added to the dish. Next, the supernatant was centrifuged at 400 × *g* for 30 min to obtain a pellet containing non‐parenchymal cells. The pellet was then mixed with 17.6% Optiprep (Axis‐Shield, 1114542), followed by the careful addition of an equal volume of 11.5% Optiprep. The mixture was centrifuged at 1400 × *g* for 40 min to enrich for LSECs and Kupffer cells, which settled in the middle layer between the 17.6% Optiprep and 11.5% Optiprep. The cell fraction was collected using a Pasteur pipette. An equal volume of PBS (containing 0.1% BSA) was added to the collected cells, followed by centrifugation at 1400 × *g* for 10 min. This washing step was repeated three times. The pelleted cells were resuspended in mouse liver sinusoidal endothelial medium and plated on cell culture dishes. After a 2‐h incubation, the supernatant containing Kupffer cells was removed, leaving behind the isolated LSECs.

### 2.5 Tube‐Formation Assay

Angiogenesis slides (ibidi, 81506) were pre‐chilled and 10 μL of uncured biogel (Millicell, ECM625) was added to each well. The slides were then incubated at 37°C for 30–45 min to allow the biogel to solidify. Meanwhile, HUVECs (1 × 10^4^) or LSECs (5 × 10^4^), previously treated with the desired reagents in the corresponding medium, were resuspended. The resuspended cells were plated onto the solidified biogel in each well and incubated at 37°C for 12 h to promote tube formation. Subsequently, the formed capillary‐like structures were visualised and captured using a microscope. For quantitative analysis, the number of tubes formed was determined by manual counting or using image analysis software such as ImageJ. Statistical analysis was performed to assess the significance of any observed differences.

### 2.6 Cell Transwell Assay

To investigate cell migration, transwell chambers (Corning, 3464) were employed. HUVECs and LSECs were subjected to overnight serum starvation. Following digestion and centrifugation, the cells were resuspended in serum‐free medium at a cell count of 4 × 10^4^ for HUVECs and 8 × 10^4^ for LSECs. The cells were then seeded onto the upper layer of the transwell chamber. Subsequently, 200 μL of serum‐free medium was added to the upper chamber, whilst 500 μL of high serum medium was added to the lower chamber. After 24 h of incubation at 37°C, the cells in the upper compartment were gently removed using a cotton swab. The migrated cells were fixed with 4% PFA for 1 h, followed by three washes with PBS. Subsequently, the cells were stained with a 1% crystal violet solution (Solarbio, C8470‐25g) for 30 min. The bottom layer of the transwell chamber was removed, and the migrated cells were mounted on a glass slide. Microscopic examination was performed, capturing four fields of view for each transwell chamber, and the number of migrated cells was counted.

### 2.7 Wound Healing Assay

To assess the migratory capacity of cells, a wound healing cell scratch migration assay was performed. HUVECs and LSECs were transfected for 48 h to achieve a cell density of 80%–90%. Cell proliferation was inhibited by treating the cells with mitomycin C (Sigma, M4287‐2mg) for 2 h. Subsequently, a sterile 10 μL pipette tip was used to create a standardised scratch on the cell monolayer. The cells were then gently washed with PBS three times to remove any detached cells. Images of the scratched area were captured at 0 h and 24 h after the scratch. To ensure the reliability of the experiment, at least three random fields of view were selected from each cell group. The distance migrated by the cells was quantitatively analysed using Photoshop software for precise assessment of their migratory capabilities.

### 2.8 Western Blotting

Protein extraction from tissue samples was performed by grinding 1 mg of tissue in liquid nitrogen, followed by the addition of 1 mL of RIPA lysis buffer (Sigma) supplemented with a protease inhibitor cocktail (100:1). The mixture was incubated with rotation at 4°C for 20 min and then centrifuged at 12,000 × *g* for 10 min to collect the supernatant. For cell protein extraction, 1 × 10^6^ cells were digested and the pellet was obtained through centrifugation. The pellet was then resuspended in 1 mL of RIPA lysis buffer containing a protease inhibitor cocktail (100:1). For phosphorylated protein detection, 2% phosphorylated protease inhibitor (Roles‐Bio, RBG2012‐1) was added to the RIPA lysis buffer. After incubation with rotation at 4°C for 20 min, the mixture was centrifuged at 12,000 × *g* for 10 min to collect the supernatant. SDS‐PAGE was performed for protein separation, with equal amounts of protein loaded into each well of the gel. Subsequently, proteins were transferred onto nitrocellulose filters (ZOMANBIO, 66485) before western blotting. A protein‐specific blocking solution (Solarbio, SW3015) was used for 1 h at room temperature to block non‐specific binding sites. Primary antibodies including NSun2 (1:1000, Abcam, ab128243), FAK (1:2000, Proteintech, 66258‐1‐lg), p‐FAK (Tyr576) (1:500, Affinity, AF3397), DUSP1 (1:500, Affinity, AF5286) and GAPDH (1:1000, Abcam, ab8245) were applied, followed by incubation with the appropriate secondary antibody. Protein content was visualised by developing on a machine.

### 2.9 Small RNA Extraction and Isolation

To perform small RNA extraction and isolation, HUVECs were transfected with *NSun2* siRNA and control siRNA to obtain small RNA fragments. A total of 5 × 10^7^ cells were collected after digestion for RNA extraction. RNA molecules with a length of less than 200 nt were extracted using the mirVana miRNA Isolation Kit (Thermo, AM1561), following the manufacturer's protocol. The extracted RNA was then subjected to electrophoresis on a 15% urea gel (acrylamide:bisacrylamide = 19:1, Thermo, EC68852BOX) containing 7 M urea in 1× TBE. Gel slices ranging from 14 to 50 nt and from 50 to 100 nt were excised based on the marker (NEB, N0364S, TaKARA, 3416). The gel slices were placed in 10 times the volume of 1 M NaCl solution, cut into smaller pieces using scissors and incubated overnight at 4°C with rotation. The supernatant containing the released RNA fragments was collected through centrifugation, followed by concentration to recover the RNA, in accordance with the instructions provided in the mirVana miRNA isolation kit. The concentration of the isolated RNA fragments was measured, and they were stored at −80°C until further use.

### 2.10 RNA Pull‐Down

To investigate the interaction between RNA and protein, we performed RNA pull‐down. First, 1‐S25 was modified with biotin to facilitate subsequent analysis. RNA pull‐down was conducted following the instructions provided with the Pierce Magnetic RNA‐Protein Pull‐Down Kit (Thermo, 20164). Then, 1 × 10^7^ HUVECs were collected and lysed to obtain at least 2 mg/mL protein lysates. Streptavidin magnetic beads were pre‐equilibrated with salt solution. 50 μL of 1× RNA Capture Buffer and biotin‐modified small RNA were mixed and incubated at room temperature for 1 h. The mixture was washed three times with 20 mM Tris buffer. Next, other reagents and protein solution were added to the washed beads. The mixture was incubated at 4°C with rotation for 2–4 h to form the RNA‐protein complex. After incubation, the beads were washed three times with 20 mM Tris buffer to remove any unbound protein. To elute the RNA‐bound protein, the beads were incubated with biotin at 37°C for 1 h. The supernatant was collected for further experiments.

### 2.11 Statistical Analysis

All statistical analyses were performed using GraphPad Prism 9 or Excel 2019. The figure legends show the statistical parameters in detail, including statistical methods, significance and *n*‐values. To determine statistical significance between samples, a *t*‐test was performed. Analysis of variance (ANOVA) was conducted for analysis of more than two groups. In the figures, statistical significance is represented as follows: n.s. denotes a non‐significant result (*p* > 0.05), * *p* < 0.05 and ** indicates *p* < 0.01.

## 3 Results

### 3.1 *NSun2* Deficiency Mitigates Liver Fibrosis via Reducing Sinusoidal Capillarisation

**FIGURE 1:**
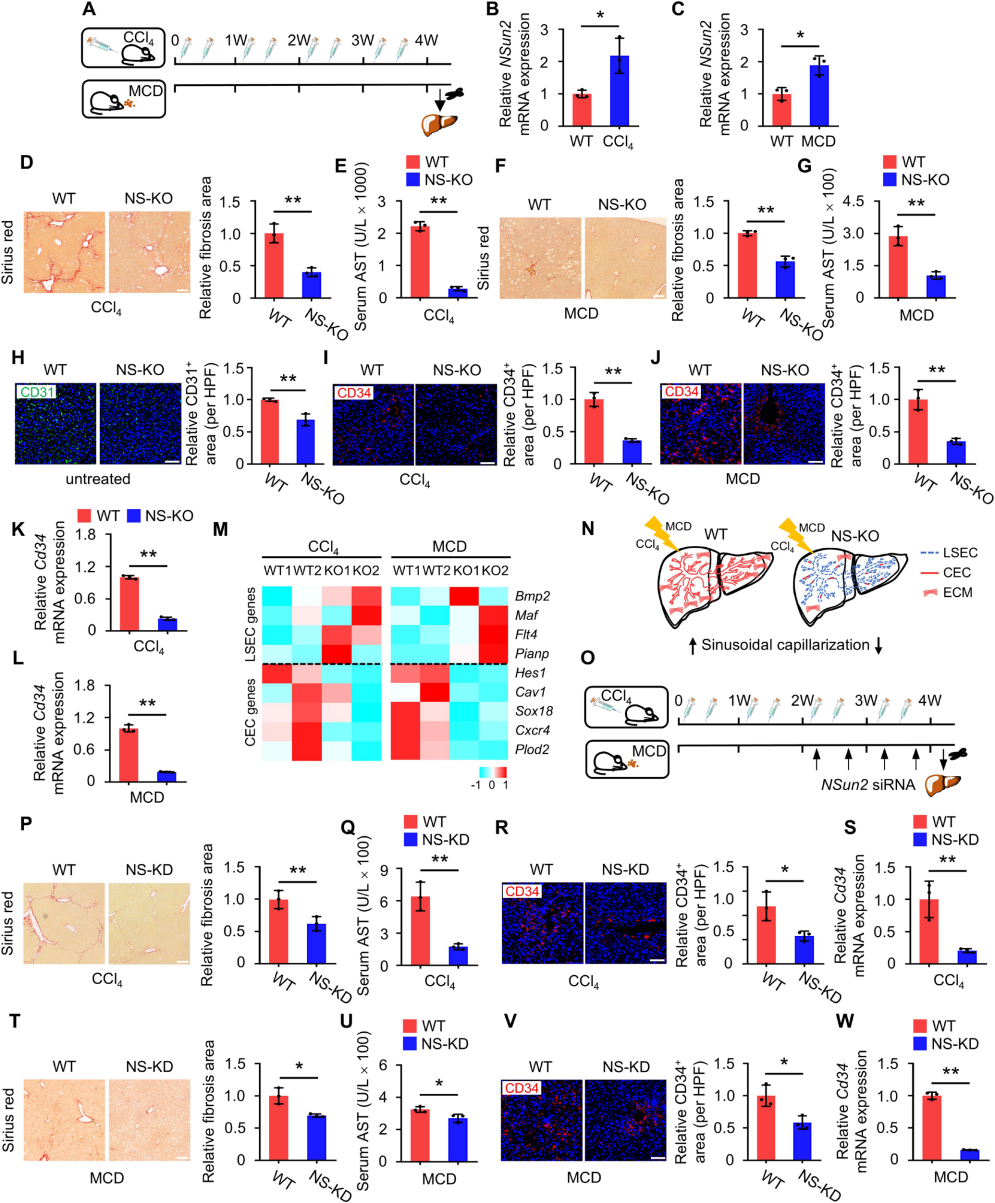
*NSun2* deficiency mitigates liver fibrosis via reducing sinusoidal capillarization. (A) Schematic diagram illustrating the induction of liver fibrosis mouse models. (B and C) The mRNA levels of *NSun2* in the livers of CCl_4_‐induced (B) and MCD‐induced (C) mouse models were compared with those of WT mice. *n* = 3 mice. (D and E) Sirius Red staining was performed on the livers of WT and NS‐KO mice after treatment with CCl_4_ (D). Serum concentrations of AST in mice were measured to determine the extent of liver injury in WT and NS‐KO mice after CCl_4_ treatment (E). The fibrotic area was quantified as a percentage. Scale bar: 100 μm; *n* = 3 mice. (F and G) Sirius Red staining was performed on the livers of WT and NS‐KO mice after treatment with MCD (F). Serum concentrations of AST in mice were measured to determine the extent of liver injury in WT and NS‐KO mice after MCD feeding (G). The fibrotic area was quantified as a percentage. Scale bar: 100 μm; *n* = 3 mice. (H) Immunofluorescence staining was performed to detect the expression of CD31 in the livers of WT and NS‐KO mice before. CD31, an endothelial cell marker, was used for visualising the liver sinusoids. Scale bar: 100 μm; *n* = 3 mice. (I and J) Immunofluorescence staining was performed to detect the expression of CD34 in the livers of WT and NS‐KO mice after treatment with CCl_4_ (I) and MCD (J). CD34, a marker for CECs, was used for visualising the sinusoidal capillarization. Scale bar: 100 μm; *n* = 3 mice. (K and L) mRNA levels of *Cd34* in the livers of WT and NS‐KO mice after treatment with CCl_4_ (K) and MCD feeding (L). *n* = 3 mice. (M) Hierarchical clustering heatmap of differentially expressed endothelial cell marker genes. (N) The schematic diagram illustrates the induction of reduced liver fibrosis in NS‐KO mice. (O) Schematic diagram illustrating the induction of liver fibrosis mouse models in NS‐KD mice. (P and Q) Sirius Red staining was performed on the livers of WT and NS‐KD mice after treatment with CCl_4_ (P). Serum concentrations of AST in mice were measured to determine the extent of liver injury in WT and NS‐KO mice after CCl_4_ treatment (Q). The fibrotic area was quantified as a percentage. Scale bar: 100 μm; *n* = 3 mice. (R and S) Assessment of sinusoidal capillarization severity was conducted in both WT and NS‐KD mice following treatment with CCl_4_. Immunofluorescence staining for CD34 was performed to quantify the presence of sinusoidal capillarization in the liver sinusoidal area (R). The mRNA levels of *Cd34* in the livers (S). Scale bar: 100 μm; *n* = 3 mice. (T and U) Sirius Red staining was performed on the livers of WT and NS‐KD mice after treatment with MCD (T). Serum concentrations of AST in mice were measured to determine the extent of liver injury in WT and NS‐KD mice after MCD feeding (U). The fibrotic area was quantified as a percentage. Scale bar: 100 μm; *n* = 3 mice. (V and W) Assessment of sinusoidal capillarization severity was conducted in both WT and NS‐KD mice following treatment with MCD. Immunofluorescence staining for CD34 was performed to quantify the presence of sinusoidal capillarization in the liver sinusoidal area (V). The mRNA levels of *Cd34* in the livers (W). Scale bar: 100 μm; *n* = 3 mice. All data are presented as mean ± SD. *p < 0.05, **p < 0.01, Student's *t*‐test.

To explore the function of *NSun2* in mouse liver fibrosis, we established two models of liver fibrosis induced by CCl_4_ injection and a MCD diet (Figures 1A and S1A). *NSun2* expression was significantly higher in fibrotic liver compared to controls (Figure 1B,C), suggesting its important role in fibrosis progression. To further explore this, we used NS‐KO mice previously generated in our lab [25] and induced liver fibrosis using CCl_4_ and the MCD diet. NS‐KO mice showed a significant reduction in collagen deposition, activated HSCs and *Col1a* expression (Figures 1D,F and S1B–D), consistent with the observed trend in serum alanine transaminase (ALT) and aspartate aminotransferase (AST) levels (Figures 1E,G and S1E). These findings suggest that NS‐KO mice exhibit attenuated liver fibrosis under the same treatment conditions as the controls.

To elucidate how *NSun2* deficiency affects fibrosis, we first examined basic liver parameters in uninjured NS‐KO mice compared to controls. We observed no significant differences in liver structure, collagen deposition, activated HSCs, Col1a expression or serum ALT and AST levels (Figure S1F–K). However, NS‐KO mice exhibited a significantly reduced density of liver sinusoids compared to controls (Figure 1H), suggesting a potential role for NSun2 in angiogenesis regulation.

During fibrosis, sinusoidal capillarization occurs, slowing substance exchange and exacerbating liver injury. The constituent cells also transform from LSECs into CECs [12]. We hypothesised that NSun2 modulates fibrosis by regulating the vascular system. We first confirmed reduced sinusoidal capillarization in the fibrotic livers of NS‐KO mice. In vitro assays assessing cluster of differentiation 34 (Cd34), a marker for CECs, revealed lower expression in NS‐KO mice (Figure 1I–L). Furthermore, mRNA‐seq analysis showed decreased expression of CEC‐specific genes in fibrotic NS‐KO livers compared with controls (Figure 1M). These results indicate that the reduced sinusoidal capillarization in NS‐KO mice contributes to the attenuation of liver fibrosis (Figure 1N).

To rule out the effect of congenital differences in liver sinusoids in the NS‐KO mice, we utilised siRNA to modulate *NSun2* expression during fibrosis (Figures 1O and S1L). *NSun2*‐knockdown (NS‐KD) mice showed reduced collagen deposition, activated HSCs and *Col1a* expression (Figures 1P,T and S1M–P), which were consistent with serum ALT and AST levels (Figures 1Q,U and S1Q,R). Additionally, markers of sinusoidal capillarization (*Cd34*) were significantly lower in NS‐KD mice compared with controls (Figure 1R,S,V,W). Collectively, these results demonstrate that *NSun2*‐knockdown inhibits sinusoidal capillarization during fibrosis.

### 3.2 *NSun2* Deficiency Impairs Endothelial Cell Migration Through tsRNA

**FIGURE 2:**
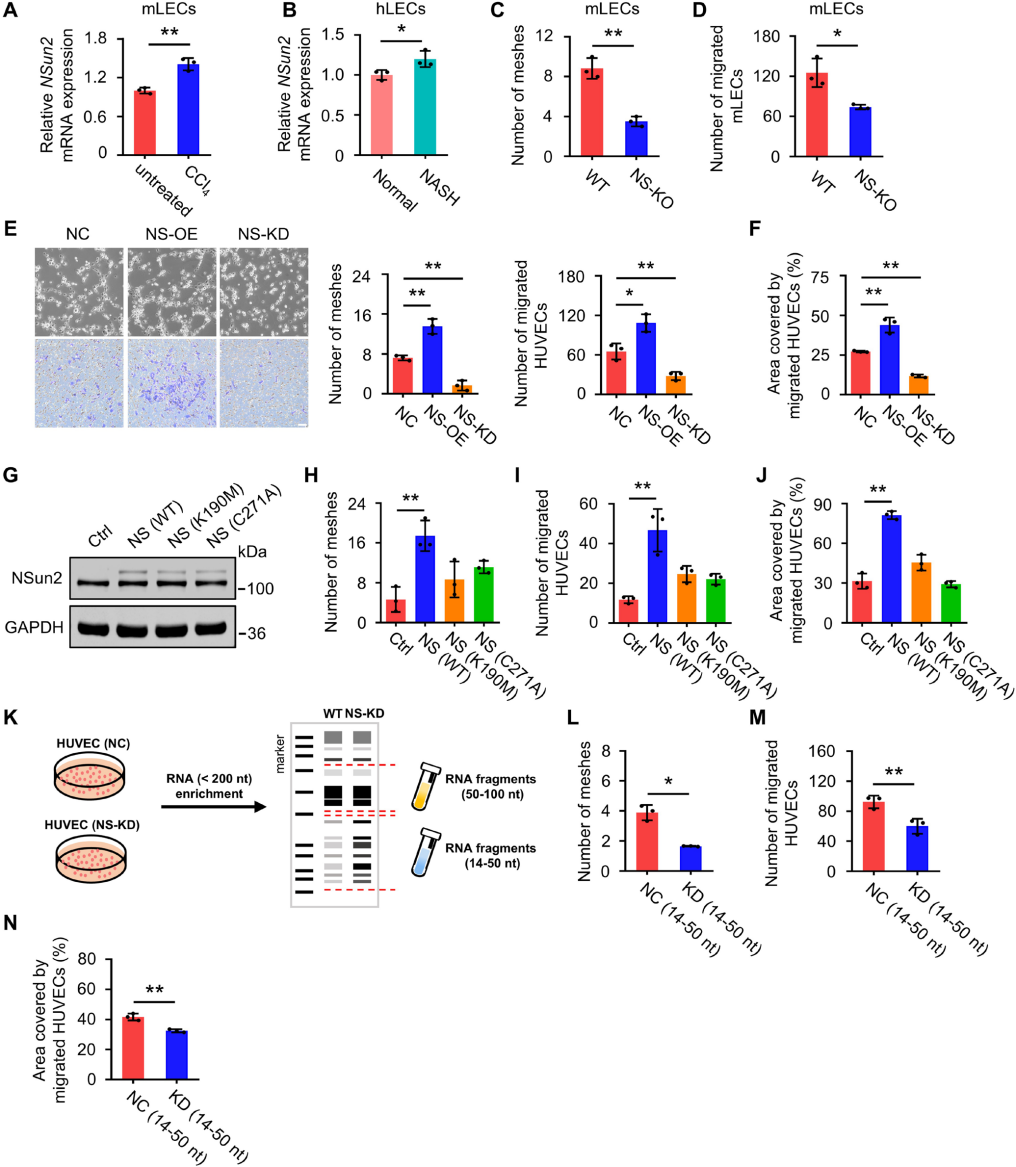
*NSun2* deficiency impairs endothelial cell migration through tsRNA. (A) The mRNA levels of *NSun2* in mLECs of WT mice and NS‐KD mice after treatment with CCl_4_. *n* = 3 mice. (B) *NSun2* expression in hLECs from normal individuals and patients with NASH. FPKM: fragments per kilobase per million mapped fragments. (C and D) Tube formation assay (C) and cell transwell assay (D) were performed to investigate the effect of *NSun2* expression on mLECs. The results of these assays are presented as statistical data. *n* = 3 replicates. (E) The effect of *NSun2* expression on tube‐forming and migratory ability in HUVECs. Left panel: representative images of tube formation and cell transwell assay are shown. Right panel: statistical results. Meshes numbers were analysed as data relative to the control, and the migrated cells were stained purple to visualise their presence. Scale bar: 100 μm; *n* = 3 replicates. (F) The effect of *NSun2* expression on migratory ability of HUVECs. The migratory ability of HUVECs was assessed using a wound healing assay, and the results are presented as statistical data. *n* = 3 replicates. (G) *NSun2*‐knockdown HUVECs were rescued with wild‐type *NSun2* (NS), two catalytically dead mutants (K190M, C271A) or an empty vector. The protein levels of NSun2 were determined in each condition. The lower band represents endogenous NSun2, whilst the upper band represents exogenous NSun2 with a flag tag. (H–J) Tube formation assay (H), cell transwell assay (I) and wound healing assay (J) were performed on *NSun2*‐knockdown HUVECs rescued by enzymatic‐dead NSun2. The results of these assays are presented as statistical data. *n* = 3 replicates. (K) Schematic for isolating small RNAs from HUVECs. Enrichment of < 200 nt RNA fragments from WT and NS‐KD HUVECs. Separation of 14–50 nt and 50–100 nt RNA fragments was performed. (L–N) Tube formation assay (L), cell transwell assay (M) and wound healing assay (N) were performed to investigate the effect of 14–50 nt small RNA derived on HUVEC. The results of these assays are presented as statistical data. *n* = 3 replicates. All data are presented as mean ± SD. *p < 0.05, **p < 0.01, Student's *t*‐test.

After confirming that *NSun2* plays a role in mitigating fibrosis through its inhibition of sinusoidal capillarization, we explored its mechanisms in vascular regulation. The rapid growth and migration of endothelial cells promote aberrant angiogenesis, which leads to sinusoidal capillarization. Additionally, cytoskeletal reorganisation during cell migration is critical for the transforming LSECs into CECs [26]. We hypothesised that the effect of *NSun2* on blood vessels might be mediated through endothelial cells. *NSun2* expression in mouse liver endothelial cells (mLECs) isolated from injured livers was significantly higher than in those from uninjured livers (Figures 2A and S2A–C). Similarly, increased *NSun2* expression was observed in human liver endothelial cells (hLECs) in NASH [27] (Figure 2B). These findings underscore NSun2's role in endothelial cells during liver fibrosis.

To further investigate the effect of NSun2 on endothelial cells, we assessed tube formation, migration and proliferation capabilities using mLECs. mLECs from NS‐KO mice showed significantly reduced tube‐forming and migratory abilities compared to controls (Figures 2C,D and S2D). Similarly, in human umbilical vein endothelial cells (HUVECs), *NSun2*‐knockdown significantly decreased, whilst its overexpression enhanced tube‐forming and migratory abilities (Figures 2E,F and S2E,F). Cell proliferation remained unaffected (Figure 2G). Therefore, impaired endothelial cell migration plays a pivotal role in compromised sinusoidal capillarisation due to *NSun2* deficiency.

Finally, we explored how NSun2, an RNA methylation enzyme, affects endothelial cell function. *NSun2* knockout results in the loss of tRNA methylation, which is crucial for the stability of tRNAs. In the absence of this modification, tRNA structures become unstable and more prone to fragmentation, increasing the levels of various tsRNAs that profoundly affect cellular functions [18]. We hypothesised that *NSun2* affects endothelial cell migration by its modification‐induced tsRNAs. NSun2 mutants K190M and C271A, which disrupt RNA modification ability, exhibited diminished tube‐forming and migration abilities compared to cells expressing normal NSun2 (Figures 2G–J and S2H,I). Importantly, cell proliferation remained unaffected (Figure S2J), indicating that *NSun2* specifically influences endothelial cell migration through RNA modification. Transfection of the 14–50 nt tsRNA fragments from NS‐KD cells significantly inhibited HUVECs tube‐forming and migratory abilities without affecting proliferation (Figures 2K–N and S2K–M). Conversely, the 50–100 nt fragments did not significantly influence cell behaviour (Figure S2N–P). These findings underscore that tsRNAs generated by reduced *NSun2* expression specifically influence endothelial cell migration, revealing a key mechanism through which NSun2 modulates sinusoidal capillarisation.

### 3.3 Identification of Two tsRNAs That Influence Cell Migration in *NSun2* ‐Knockdown HUVECs

**FIGURE 3:**
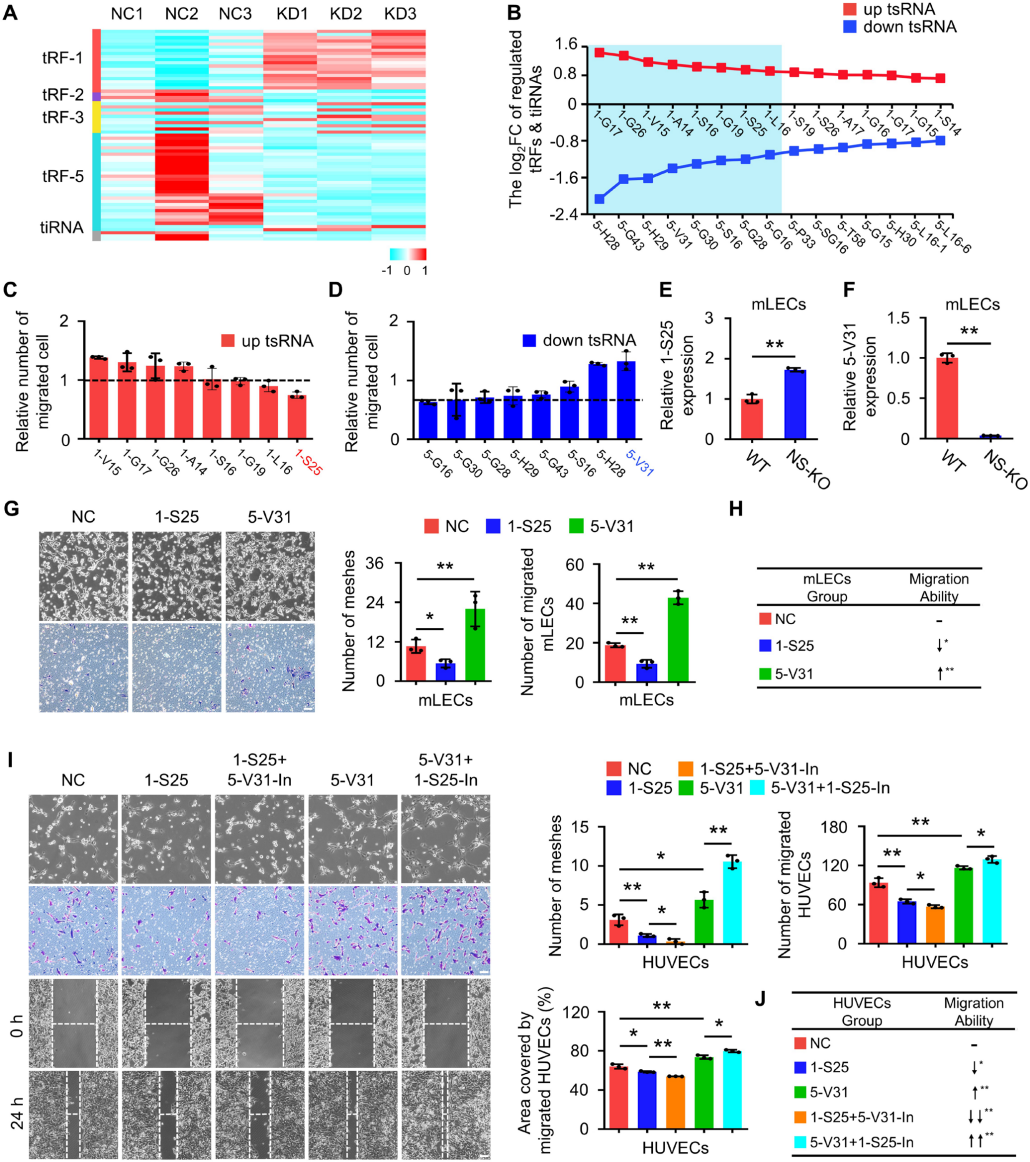
Identification of two tsRNAs that influence cell migration in *NSun2*‐knockdown HUVECs. (A) Heatmap showing differential expression of tsRNAs. (B) Altered tsRNAs were depicted. Red lines represent upregulated tsRNAs, whilst blue lines indicate downregulated tsRNAs. The top eight tsRNAs for each trend are labelled in azure. (C and D) Selection of tsRNA based on migratory ability using cell transwell assay for the top eight upregulated (C) and downregulated (D) tsRNAs. *n* = 3 replicates. (E and F) The expression levels of 1‐S25 (E) and 5‐V31 (F) in WT and NS‐KO primary mLECs. *n* = 3 replicates. (G) The effects of 1‐S25, 5‐V31 and their combination on tube‐forming and migratory ability of mLECs were evaluated using the tube formation assay and cell transwell assay. Scale bar: 100 μm; *n* = 3 replicates. (H) The chart illustrates how different groups of small RNA, namely, NC small RNA, 1‐S25, 5‐V31, impact mLECs migration ability. An upward arrow indicates promotion of cell migratory ability, whilst a downward arrow indicates inhibition of cell migratory ability. (I) The effects of 1‐S25, 5‐V31 and their combination on the tube‐forming and migratory ability of HUVECs were evaluated using the tube formation assay, cell transwell assay and wound healing assay. The results of these assays were analysed statistically, and both quantitative data and representative images are presented to illustrate the outcomes. Scale bar: 100 μm; *n* = 3 replicates. (J) The chart illustrates how different groups of small RNA, namely, NC small RNA, 1‐S25, 5‐V31, 1‐S25 with 5‐V31‐In and 5‐V31 with 1‐S25‐In, impact HUVECs migration ability. An upward arrow indicates promotion of cell migratory ability, whilst a downward arrow indicates inhibition of cell migratory ability. All data are presented as mean ± SD. *p < 0.05, **p < 0.01, Student's *t*‐test.

*NSun2*‐knockdown in endothelial cells produces tsRNAs that are capable of inhibiting cell migration. To identify tsRNAs influencing migration, we performed tRF and tiRNA‐seq, which revealed significant changes in tRF‐1 and tRF‐5 (Figure 3A). Next, we selected 16 small RNAs with the largest log_2_FC variations for further analysis (Figure 3B and Table S1). Amongst these, tRF‐1‐S25 (1‐S25) and tRF‐5‐V31 (5‐V31) demonstrated the most significant impacts on cell migration, and their expression levels were validated in vitro using NS‐KD HUVECs (Figures 3C,D and S3A,B). Next, we also demonstrated the regulatory effects of 1‐S25 and 5‐V31 on cell migration in mLECs from NS‐KO mice (Figures 3E–H and S3C,D). These findings suggest that 1‐S25 and 5‐V31 are key players in the influence of NSun2 on cell migration.

To explore their cooperative effects in cell migration, we observed that, compared to the 1‐S25 alone, the combined 1‐S25 and 5‐V31 inhibitor (5‐V31‐In) led to a greater reduction in tube‐forming and migratory abilities. Conversely, co‐transfection with the 1‐S25 inhibitor (1‐S25‐In) and 5‐V31 enhanced these abilities compared to 5‐V31 alone (Figures 3I,J and S3E,F). Importantly, cell proliferation remained unaffected (Figure S3G). Furthermore, restoration experiments after *NSun2* knockdown confirmed that NSun2 exerts its effects through these tsRNAs (Figure S3H–M). In conclusion, our analysis identified two tsRNAs from NS‐KD cells that significantly impact endothelial cell migration. Moreover, these two small RNAs exhibited a synergistic effect on cell behaviour.

### 3.4 The Mechanism by Which the Two tsRNAs Regulate Cell Migration

**FIGURE 4:**
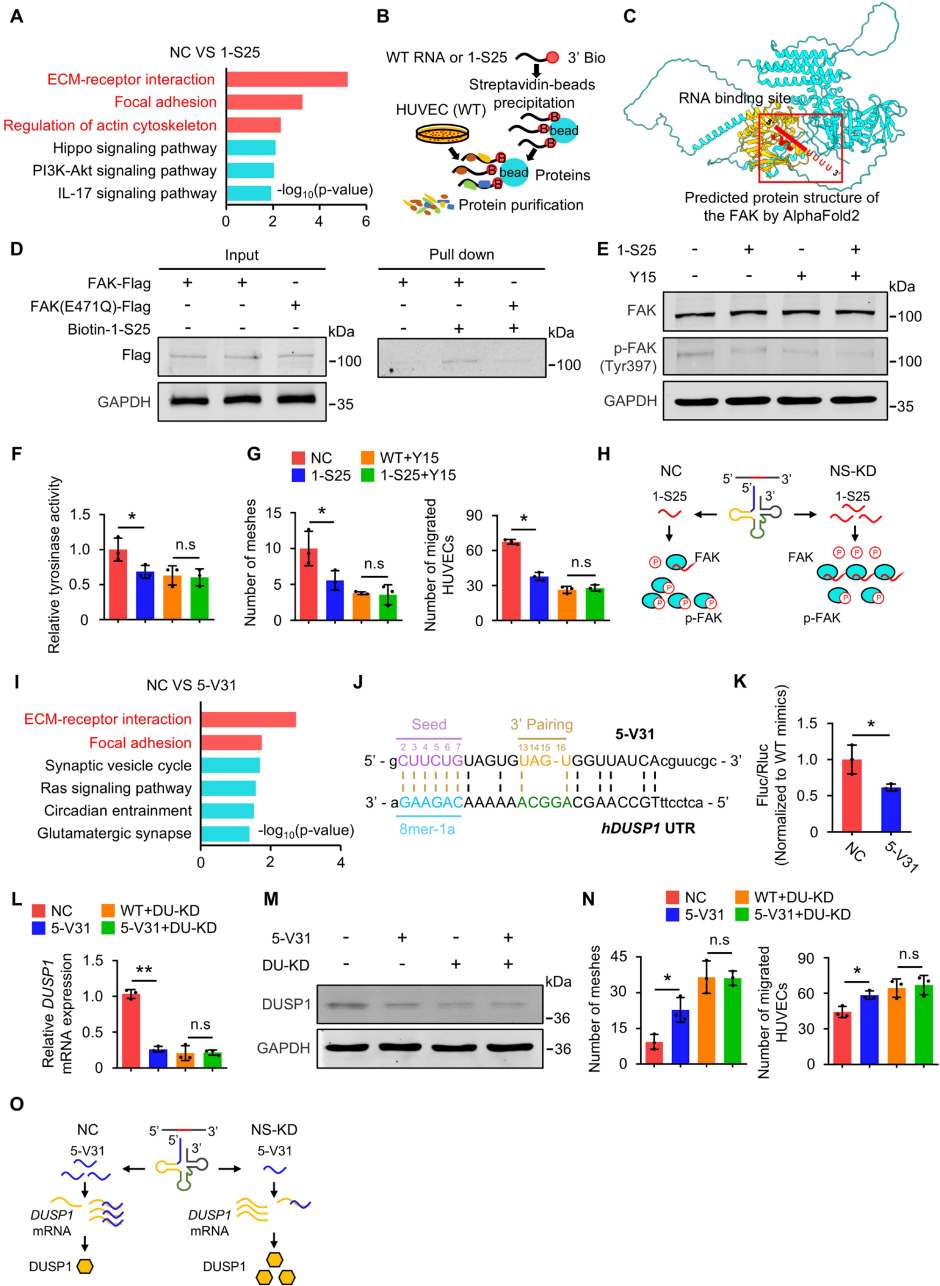
The mechanism by which the two tsRNAs regulate cell migration. (A) KEGG analysis of differentially expressed genes between WT‐ and 1‐S25‐transfected HUVECs. (B) The schematic of using RNA pull‐down to screen interactive proteins with 1‐S25. (C) Predicted binding site of 1‐S25 at FAK protein. (D) RNA pull‐down experiments verified the binding site of 1‐S25 at FAK protein in HUVECs. (E) The functional role of 1‐S25 was validated by inhibiting FAK phosphorylation. The protein levels of FAK and phosphorylated p‐FAK (Tyr397) in HUVECs were determined in each experimental condition. Y15 is a FAK phosphorylation inhibitor. (F) The tyrosinase activity of FAK was assayed. *n* = 3 replicates. (G) Tube formation assay and cell transwell assay of FAK were performed in 1‐S25‐transfected HUVECs in which FAK was inhibited. The results of these assays were analysed statistically; *n* = 3 replicates. (H) Mechanism of action of 1‐S25. (I) KEGG analysis of differentially expressed genes between WT‐ and 5‐V31‐transfected HUVECs. (J) Predicted 5‐V31‐binding site at the 3′UTR of human *DUSP1* mRNA. (K) Dual luciferase assay confirms 5‐V31 binding at human *DUSP1* mRNA 3′UTR. (L and M) The functional role of 5‐V31 was validated by inhibiting DUSP1 expression. The mRNA (L) and protein (M) levels of DUSP1 in HUVECs were determined in each experimental condition. *n* = 3 replicates. (N) Tube formation assay and cell transwell assay were performed in 5‐V31‐transfected HUVECs in which *DUSP1* was knocked down. The results of these assays were analysed statistically. Scale bar: 100 μm; *n* = 3 replicates. (O) Mechanism of action of 5‐V31. All data are presented as mean ± SD. *p < 0.05, **p < 0.01, Student's *t*‐test.

We explored how the two tsRNAs influence cell function. First, we analysed 1‐S25. mRNA‐seq analysis revealed that 1‐S25 regulates pathways associated with the actin cytoskeleton, correlating with cell migratory ability (Figure 4A). Then, mRNA target prediction for 1‐S25 did not identify the expected genes (Table S2). Next, RNA pull‐down assays revealed substantial enrichment of FAK protein (Figure 4B and Table S4). FAK, a tyrosine kinase, is a crucial modulator of the actin cytoskeleton. Phosphorylated FAK modulates cell migration and influences capillarization [28]. We identified a 1‐S25 binding site in the phosphorylation region of FAK and validated this interaction using a mutant FAK (E471Q) (Figure 4C,D). Additionally, this binding site showed 100% similarity between mice and humans (Figure S4A). We propose that 1‐S25 inhibits FAK phosphorylation, affecting cell behaviour (Figure 4D). Results revealed a significant reduction in p‐FAK (Tyr397) following 1‐S25 transfection, with unchanged total FAK levels (Figures 4E and S4B). 1‐S25 also decreased FAK tyrosine kinase activity (Figures 4F and S4C,D). Y15, a FAK phosphorylation inhibitor, abolished both the effect of 1‐S25 on p‐FAK and cellular phenotype (Figures 4E,G and S4E–G). These findings firmly confirm that 1‐S25 modulates cell migration by inhibiting FAK phosphorylation (Figure 4H).

Next, we analysed the mechanisms by which 5‐V31 affects endothelial cells. mRNA‐seq data from HUVECs transfected with 5‐V31 revealed similar findings to those observed with 1‐S25 transfection (Figure 4I). mRNA target prediction for 5‐V31 identified dual specificity phosphatase‐1 (DUSP1), whose downregulation promotes cell migration [29] (Table S3). The binding of 5‐V31 to the *DUSP1* mRNA 3′UTR was validated in both humans and mice (Figures 4J,K and S4H). We hypothesised that 5‐V31 modulates cell migration by regulating *DUSP1*. Experiments showed significantly reduced DUSP1 levels following 5‐V31 transfection. *DUSP1* siRNA abolished the effect of 5‐V31 (Figures 4L,M and S4I). Furthermore, *DUSP1* siRNA also abolished the effect of 5‐V31 on cellular phenotype (Figures 4N and S4J–L). These findings indicate that 5‐V31 primarily regulates cell migration by modulating *DUSP1* expression (Figure 4O).

### 3.5 The Synergistic Effect of Two tsRNAs on FAK in the Regulation of Cell Migration

After confirming the specific targets of the two tsRNAs, we explored their synergy. Specifically, *DUSP1*, regulated by 5‐V31, dephosphorylates extracellular regulated protein kinases (ERK) [30], and phosphorylated ERK promotes cell migration by targeting FAK [31], regulated by 1‐S25. Therefore, we hypothesised that these tsRNAs synergistically influence FAK phosphorylation.

**FIGURE 5:**
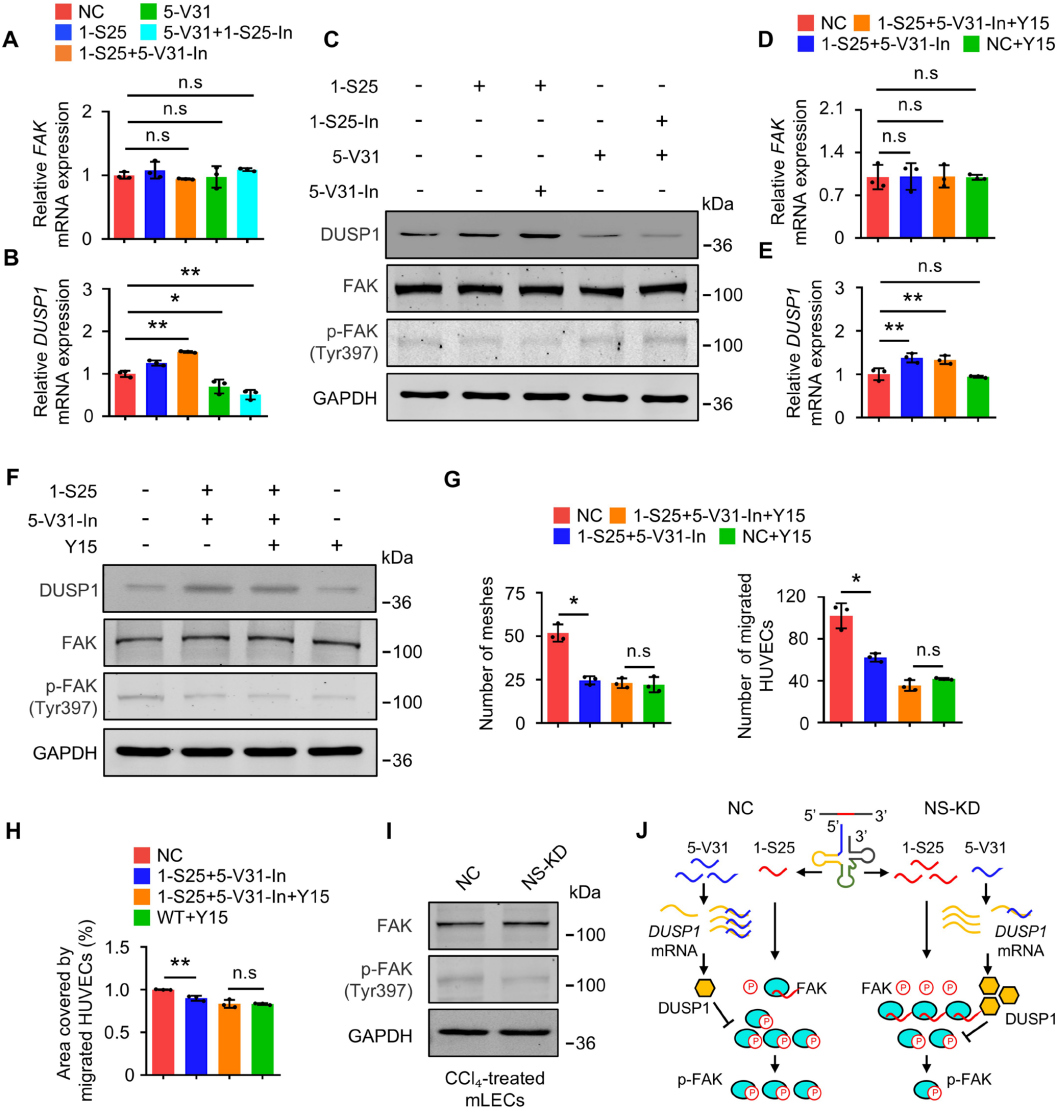
The synergistic effect of the two tsRNAs on FAK in the regulation of cell migration. (A and B) HUVECs were used to validate the effects of 1‐S25, 5‐V31 and their combination on cellular functionality. The mRNA levels of *FAK* (A) and *DUSP1* (B) were determined in each experimental condition. *n* = 3 replicates. (C) The effects of 1‐S25, 5‐V31 and their combination on the FAK phosphorylation of HUVECs were evaluated using western blotting assay. (D and E) The collaborative functional role of 1‐S25 and 5‐V31 was validated by inhibiting FAK phosphorylation. The mRNA expression levels of *FAK* (D) and *DUSP1* (E) in HUVECs were determined in each experimental condition. *n* = 3 replicates. (F) The collaborative functional role of 1‐S25 and 5‐V31 was validated by inhibiting FAK phosphorylation. The protein levels of DUSP1, FAK and phosphorylated p‐FAK (Tyr397) in HUVECs were assessed under each experimental condition. (G) Tube formation assay and cell transwell assay of FAK was performed in 1‐S25‐ and 5‐V31‐In‐transfected HUVECs in which FAK was inhibited. The results of these assays were analysed statistically, and both quantitative data are presented to illustrate the outcomes. *n* = 3 replicates. (H) Wound healing assay was performed in 5‐V31‐In transfected HUVECs in which FAK was inhibited. The results of these assays were analysed statistically, and both quantitative data are presented to illustrate the outcomes. *n* = 3 replicates. (I) The effect of NSun2 on FAK phosphorylation in mLECs under CCl_4_ treatment was evaluated by western blotting. (J) Schematic for the mechanism of action of 1‐S25 and 5‐V31, both of which can collaboratively target FAK, influencing cellular functions. All data are presented as mean ± SD. *p < 0.05, **p < 0.01, Student's *t*‐test.

We found that FAK‐Tyr397 levels were significantly reduced in the 1‐S25 and 5‐V31‐In groups compared to controls, whilst FAK levels were unchanged (Figure 5A–C). Adding Y15 to the 1‐S25 and 5‐V31‐In co‐transfection groups showed no significant difference in p‐FAK (Tyr397) levels compared to the only Y15 group. Meanwhile, *DUSP1* expression increased in the 1‐S25 and 5‐V31‐In co‐transfection group (Figures 5D–F and S5A,B), indicating that the regulation of p‐FAK by DUSP1 was inhibited. Phenotypically, transfection with the 1‐S25 and 5‐V31‐In after the addition of Y15 did not affect tube formation and migratory ability (Figures 5G,H and S5C,D). Cell proliferation remained unaffected (Figure S5E). These results indicate that FAK is the key regulatory target of the two tsRNAs, which synergistically affect its phosphorylation.

We plan to further investigate whether the tsRNA‐mediated changes in FAK phosphorylation observed in vitro also occur in endothelial cells in fibrotic mice upon *NSun2* deletion. To this end, we first isolated mLECs and performed *NSun2* knockdown, followed by treatment with CCl_4_ to mimic the stress conditions encountered by endothelial cells in the murine liver fibrosis model. Experimental results showed that *NSun2* deficiency significantly reduced FAK phosphorylation levels in mLECs (Figure 5I and S5F). These findings confirm that *NSun2* deletion in endothelial cells within fibrotic mouse livers also leads to altered FAK phosphorylation. This result clarifies the molecular mechanism by which NSun2 regulates FAK phosphorylation in vivo through the modulation of tsRNAs expression, thereby influencing cellular function (Figure 5J).

### 3.6 Amelioration of Liver Fibrosis in Mice by the tsRNAs and Analysis of Public Liver Disease Data in Humans

**FIGURE 6:**
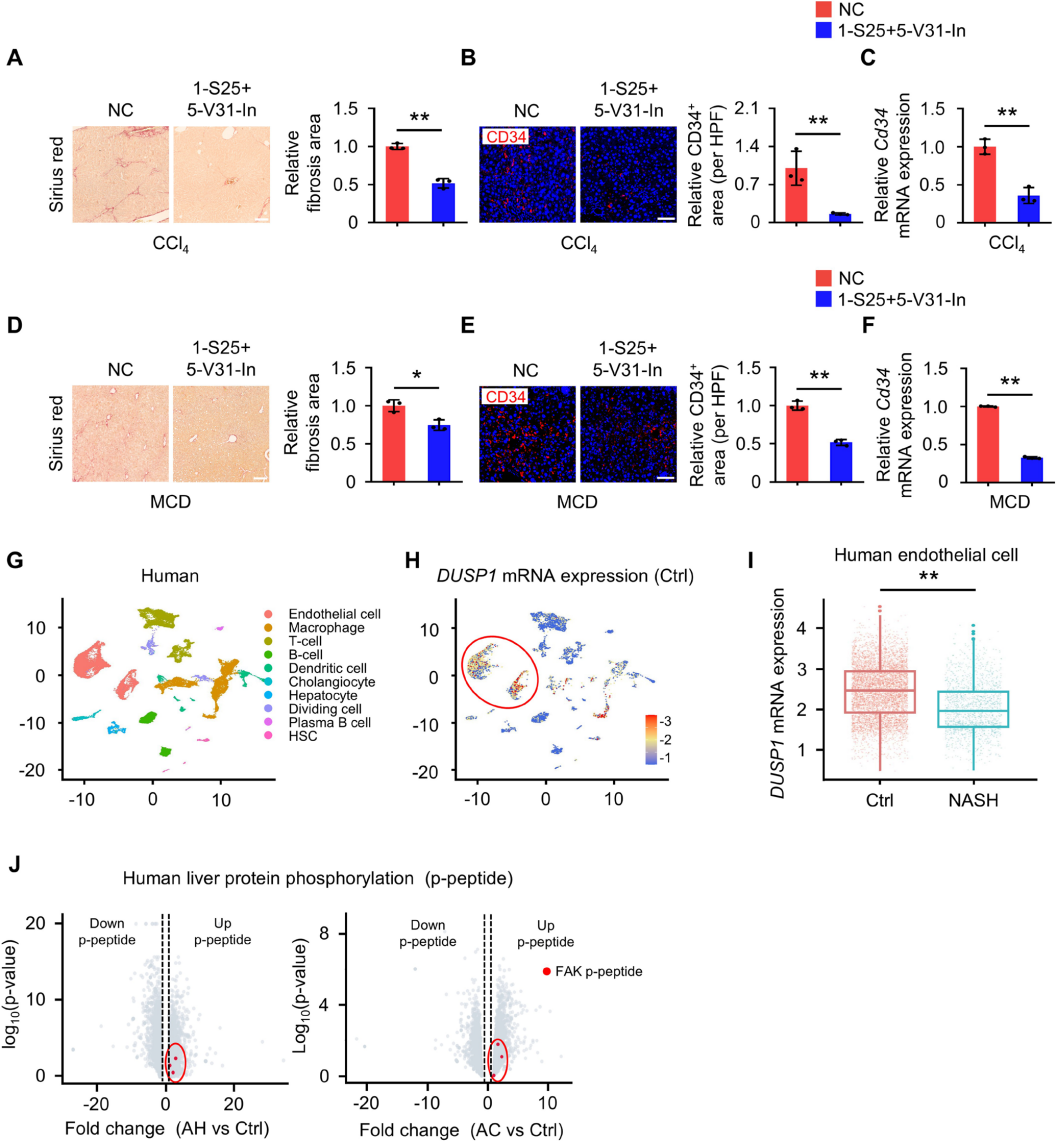
Amelioration of liver fibrosis in mice by the two tsRNAs and analysis of public liver disease data in humans. (A) The severity of liver injury was assessed in a CCl_4_‐induced liver fibrosis mouse model after injection with NC small RNA or 1‐S25 + 5‐V31‐In. Sirius Red staining was performed on the livers to quantify the presence of fibrotic area. Scale bar: 100 μm; *n* = 3 mice. (B and C) Immunofluorescence staining was performed to detect the expression of CD34 in the livers of mice with CCl_4_‐induced liver fibrosis after injection with NC small RNA or 1‐S25 + 5‐V31‐In (B). The mRNA levels of *Cd34* in the livers (C). *Cd34*, the marker of sinusoidal capillarization. Scale bar: 100 μm; *n* = 3 mice. (D) The severity of liver injury was assessed in a liver fibrosis mouse model induced by feeding MCD, after injection with NC small RNA or 1‐S25 + 5‐V31‐In. Sirius Red staining was performed on the livers to quantify the presence of fibrotic area. Scale bar: 100 μm; *n* = 3 mice. (E and F) Immunofluorescence staining was performed to detect the expression of CD34 in the MCD‐induced liver fibrosis mouse model after injection with NC small RNA or 1‐S25 + 5‐V31‐In (E). The mRNA levels of *Cd34* in the livers (F). Scale bar: 100 μm; *n* = 3 mice. (G) Clustering of non‐parenchymal cells in liver tissues from healthy individuals and patients with NASH, using single‐cell RNA sequencing. (H) Expression level of *DUSP1* in non‐parenchymal cells. (I) Expression level of *DUSP1* in human endothelial cells from healthy individuals and patients with NASH. (J) Liver protein phosphorylation sequencing in normal individuals with alcoholic hepatitis (AH) and alcoholic cirrhosis (AC). Red dots indicate phosphorylation sites of FAK. All data are presented as mean ± SD. *p < 0.05, **p < 0.01, Student's *t*‐test.

After establishing the in vitro action of the two tsRNAs, we investigated their therapeutic efficacy in liver fibrosis using two mouse models (Figure S6A–C). Treatment with 1‐S25 and 5‐V31‐In significantly reduced collagen deposition, activated HSCs, *Col1a* expression and AST levels (Figures 6A,D and S6D,E,G–I). Other serum parameters remained unchanged (Figure S6F). Interestingly, sinusoidal capillarization was significantly reduced in the RNA‐injected group compared to controls (Figure 6B,C,E,F). These findings suggest that the two tsRNAs inhibit sinusoidal capillarization, alleviating liver fibrosis.

To validate the relevance of our findings in humans, we analysed single‐cell data from reported human liver injury samples [27] (Figure 6G). *DUSP1* was highly expressed in endothelial cells of control samples (Figure 6H), but significantly downregulated in NASH injury samples (Figure 6I). Additionally, phosphoproteomics data from published alcohol‐associated hepatitis (AH) and alcohol‐associated cirrhosis (AC) injury samples showed a significant increase in FAK phosphorylation following injury [32] (Figure 6J). The targets of the two small RNAs in human liver injury samples change consistent with RNA regulation. Thus, the two tsRNAs may also have a therapeutic role in human liver injury and fibrosis.

## 4 Discussion

Here, we show that *NSun2* deficiency in mice ameliorates liver fibrosis by reducing sinusoidal capillarization. Two tsRNAs produced as a result of *NSun2* deficiency, namely, 1‐S25 and 5‐V31, play crucial roles in this process. 1‐S25 directly regulates the phosphorylation of FAK, whilst 5‐V31 indirectly affects FAK phosphorylation by influencing *DUSP1*. Together, they synergistically inhibit the phosphorylation of FAK, a key regulatory factor in endothelial cell angiogenesis. Injection of 1‐S25 and 5‐V31‐In into a liver fibrosis mouse model effectively alleviated sinusoidal capillarization and liver fibrosis, highlighting their potential therapeutic role in liver injury as well.

Liver fibrosis is a crucial target for therapeutic intervention in liver disease. However, current treatment options for severe liver fibrosis are limited, with donor organ transplantation being the primary effective method. tsRNAs are newly discovered functional small RNAs with significant therapeutic potential in various disorders, including liver diseases [33, 34]. For instance, NSun2 generates tsRNAs that accelerate recovery from acute liver injury [25]. Additionally, the inhibition of specific tsRNA Gly‐tRFs in vivo has been shown to decrease hepatosteatosis induced by chronic consumption of ethanol [35]. Furthermore, the vascular system is a critical target in liver fibrosis treatment. Existing literature supports that tsRNAs are key regulators of endothelial cell behaviour in angiogenesis [36]. A previous study reports that the products of tsRNAs cleavage by angiogenin, such as tRNAVal and tRNAGly‐derived fragments, hinder cell proliferation, migration and tube formation [37]. This further demonstrates the close relationship between tsRNAs and liver fibrosis. However, the precise role of tsRNAs in liver fibrosis remains largely unexplored. This study first identifies two tsRNAs – 1‐S25 and 5‐V31 – whose levels are modulated by changes in NSun2 expression and have opposing functions. Upregulating 1‐S25 whilst downregulating 5‐V31 synergistically alleviates liver fibrosis by modulating sinusoidal capillarization. This finding indicates a novel therapeutic approach for fibrosis treatment.

Sinusoidal capillarization is closely related to the progression of liver fibrosis. However, despite the demonstrated anti‐fibrotic effects of numerous drugs targeting blood vessels in animal models, these approaches have not translated well to clinical applications [38]. This is partly because current vascular targets primarily focus on the VEGF signalling pathway. Unlike other vascular diseases, VEGF has paradoxical roles in liver fibrosis; on one hand, inhibition of VEGF receptors in vivo can reduce fibrosis formation and portal hypertension [39]. On the other hand, the inhibition of VEGF receptors interferes with the resolution of fibrosis mediated by matrix metallopeptidase 13 (MMP13) [40]. In our study, FAK, as an intracellular cytoskeletal regulator in endothelial cells, modulates actin aggregation at cellular protrusions by interfering with intracellular cytoskeletal remodelling, thereby regulating cell migration and playing a role in sinusoidal capillarization. FAK targets, unlike VEGF, have not shown conflicting functions [28]. Our study showed that targeting FAK phosphorylation by tsRNAs can selectively regulate sinusoidal capillarization without eliciting conflicting functions similar to those of VEGF.

Current therapeutic approaches targeting FAK phosphorylation primarily depend on small molecule inhibitors, such as IN10018, defactinib, conteltinib and APG‐2449 [41]. However, these small molecules encounter significant challenges, including difficulties in achieving specific delivery, the development of resistance with prolonged use and the potential for severe side effects. Consequently, there is an urgent need for alternative treatment strategies. In this context, small RNA therapy represents a promising advancement, as it circumvents many limitations associated with small molecule drugs, offering enhanced compatibility, therapeutic efficacy and precise targeting [42, 43]. By utilising specific AAV viral vectors or nanoparticle delivery systems, small RNAs can achieve cell or tissue‐specific targeting, such as in the liver or different regions of the brain, making them suitable for long‐term treatment whilst minimising side effects [44, 45]. Nevertheless, small RNA molecules specifically designed to target FAK phosphorylation have yet to be identified. Notably, tsRNAs demonstrate the potential to regulate proteins; for instance, Glu‐5'tsRNA‐CTC has been reported to interfere with the function of leucyl‐tRNA synthetase 2 (LaRs2), thereby inhibiting mitochondrial protein translation [46]. In our study, we first identified and validated the regulatory capabilities of two types of natural tsRNAs on FAK phosphorylation, underscoring the potential of small RNA modulation. Furthermore, these two tsRNAs exhibit synergistic interactions that enhance their regulatory efficacy. It is anticipated that these tsRNAs may have potential as targeted drugs for FAK.

In this study, tsRNAs treatment demonstrated therapeutic effects in both CCl_4_‐and MCD diet‐induced liver fibrosis models, although its efficacy was notably lower in the CCl_4_ model. We speculate that this discrepancy arises from differences in disease progression and the timing of tsRNAs administration. By the third week of CCl_4_ exposure, LSECs exhibit reduced fenestration but have not yet undergone full capillarization – a transitional state that may limit the therapeutic effectiveness of angiogenesis‐targeting tsRNAs [9]. In contrast, by the third week of MCD diet‐induced fibrosis, LSECs have undergone complete capillarization and exhibit a mature, capillary‐like phenotype [47, 48]. Since tsRNAs treatment was initiated at the third week in both models, it likely coincided with an optimal therapeutic window in the MCD model but was administered too early in the CCl_4_ model. These findings highlight the importance of disease aetiology and temporal progression in defining effective therapeutic windows for RNA‐based interventions.

The vasculature is a prominent therapeutic target in liver fibrosis [49]. However, existing clinical outcomes from targeting the vasculature have been disappointing. In addition to optimising target selection for vascular treatments, a new approach has been proposed in recent years. This approach focuses on more precise targeting of vascular locations within the hepatic lobule. The hepatic lobule vascular system comprises the portal vessels, central vein vessels and hepatic sinusoids [50]. Research proposes that portal angiogenesis during fibrosis contributes to the delivery of oxygen and nutrients to hepatic sinusoids, thereby facilitating fibrosis recovery [51]. Therefore, in addition to inhibiting the growth and capillarisation of central vein vessels and hepatic sinusoids, targeting portal vessel markers such as leukocyte cell‐derived chemotaxin‐2 (LECT2) to promote portal vessel growth can more effectively reduce liver fibrosis, outperforming the sole inhibition of capillarisation [52]. In our experiments, unintentional inhibition of normal portal vessel growth occurred because of the broad targeting range of the tsRNAs (Figure 6B,E), potentially hindering the treatment of liver fibrosis. Thus, in subsequent research, we will attempt to combine the use of a viral vector promoting portal vessel growth with the two functional tsRNAs identified in our study. This combined approach holds the potential for superior therapeutic effects compared to the use of either tsRNA alone.

Overall, we propose that targeting sinusoidal capillarisation through the modulation of FAK phosphorylation via the synergistic action of the two tsRNAs represents a novel therapeutic target for the treatment of liver fibrosis. These contributions are likely to spur further research and potential clinical applications, leading to the development of more effective treatments for liver fibrosis.

## Author Contributions

Qi Zhou, Wei Li and Ying Zhang: conceptualisation. Pengcheng Li, Sunyang Ying, Yu Zou, Runxue Zhang, Cheng Huang, Moyu Dai and Haiping Jiang: methodology. Xin Wang, Pengcheng Li, Xin Li, Zhikun Li and Guihai Feng: formal analysis. Kai Xu: resources. Pengcheng Li, Sunyang Ying, Yu Zou and Xin Wang: writing – original draft. Ying Zhang and Wei Li: writing – review and editing.

## Conflicts of Interest

The authors declare no conflicts of interest.

## Supporting information


**Data S1.** Supporting information.

## Data Availability

Single‐cell sequencing data from patients with non‐alcoholic steatohepatitis (NASH) and healthy individuals were obtained from the Gene Expression Omnibus (GEO) under accession number GSE129516. The tsRNAs sequencing profiling data and raw data have been uploaded to Genome Sequence Archive (GSA) under the following accession number: HRA008098. The RNA‐seq data have also been deposited in GSA under the accession number CRA017831. The mass spectrometry and proteomics data have been deposited in the ProteomeXchange Consortium (http://proteomecentral.proteomexchange.org) via the iProX partner repository with the dataset identifier PXD054259.
