# Supplementary material for: NSun2‐Mediated tsRNAs Alleviate Liver Fibrosis via FAK Dephosphorylation
Source: Cell Prolif. 2025 May 12;58(10):e70058. doi: 10.1111/cpr.70058 (PMC12508683; doi:10.1111/cpr.70058)
Supplement: Supplementary file 1 — Data S1. Supporting information. [file CPR-58-e70058-s001.docx]

**Supplementary Figure and Figure legends**


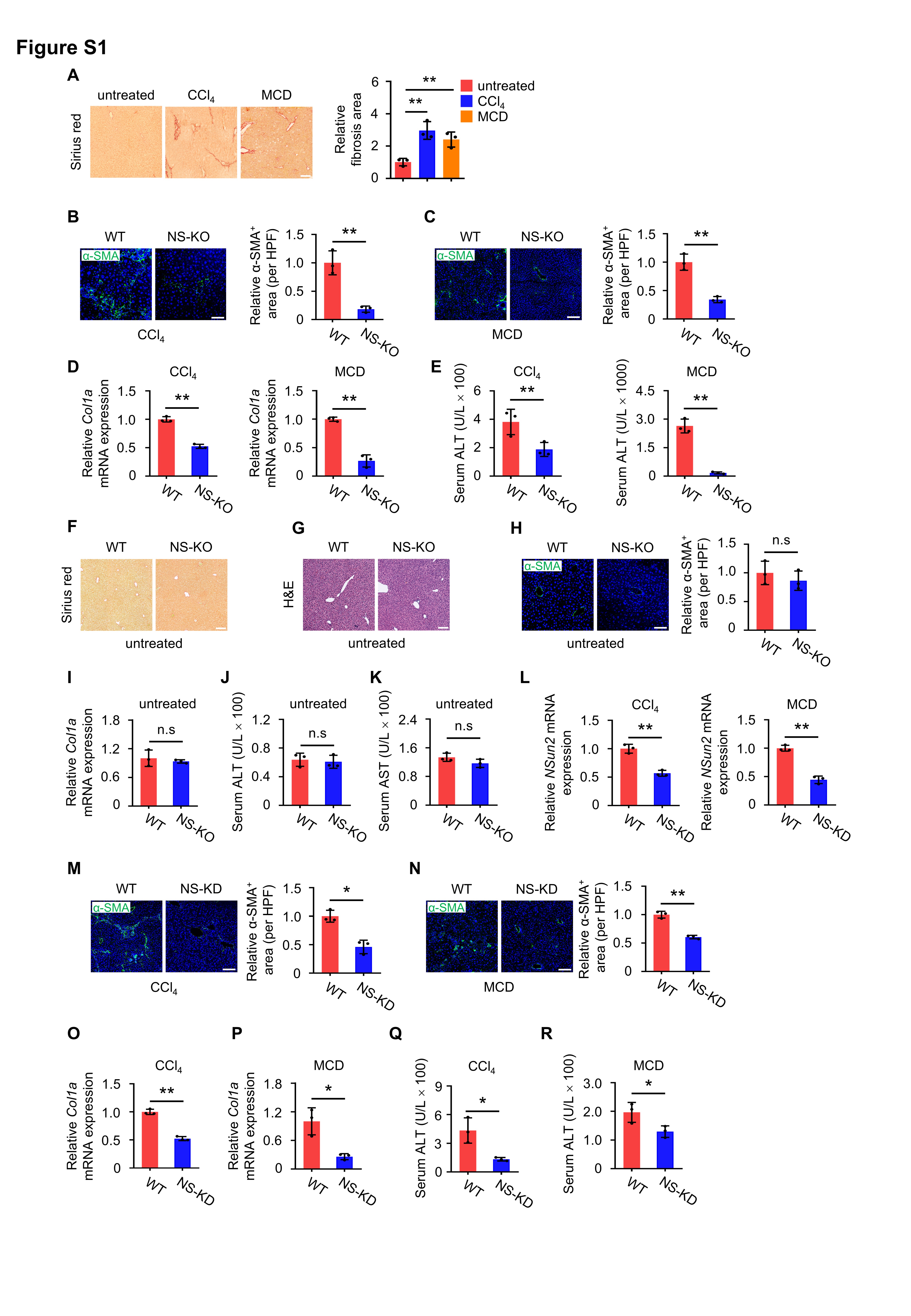


**Figure S1. Normal liver phenotype in *NSun2*-deficient mice and attenuation of fibrosis with NSun2 knockdown during injury.**

(A) Sirius Red staining was performed on liver sections. Scale bar: 100 μm; n = 3 mice. (B, C) Immunofluorescence staining was performed to detect the expression of α-SMA in the livers of WT and NS-KO mice after treatment with CCl_4_ (B) and MCD feeding (C). Scale bar: 100 μm; n = 3 mice. (D) The mRNA levels of *Col1a* in the livers. n = 3 mice. (E) Mouse serum ALT concentrations was assessed to determine the degree of liver injury, n = 3 mice. (F) Sirius Red staining in the livers of WT and NS-KO mice. Scale bar: 100 μm; n = 3 mice. (G) Hematoxylin and eosin staining in the livers of WT and NS-KO mice. Scale bar: 100 μm; n = 3 mice. (H) Immunofluorescence staining was performed to detect the expression of α-SMA in the livers. Scale bar: 100 μm; n = 3 mice. (I) The mRNA levels of *Col1a* in the livers. (J, K) Mouse serum ALT (J) and AST (K) concentrations were assessed to determine the degree of liver injury. n = 3 mice. (L) The mRNA levels of *NSun2* in the livers. (M, N) Immunofluorescence staining was performed to detect the expression of CD31 in the livers of WT and NS-KD mice after treatment with CCl_4_ (M) and MCD (N), Scale bar: 50 μm; n = 3 mice. (O, P) The mRNA levels of *Col1a* in the livers of WT and NS-KD mice after treatment with CCl_4_ (O) and MCD (P). (Q, R) Mouse serum ALT concentration was assessed to determine the degree of liver injury in WT and NS-KD mice after treatment with CCl_4_ (Q) and MCD (R). n = 3 mice. All data are presented as mean ± SD. **p* < 0.05, ***p* < 0.01, Student’s *t*-test.


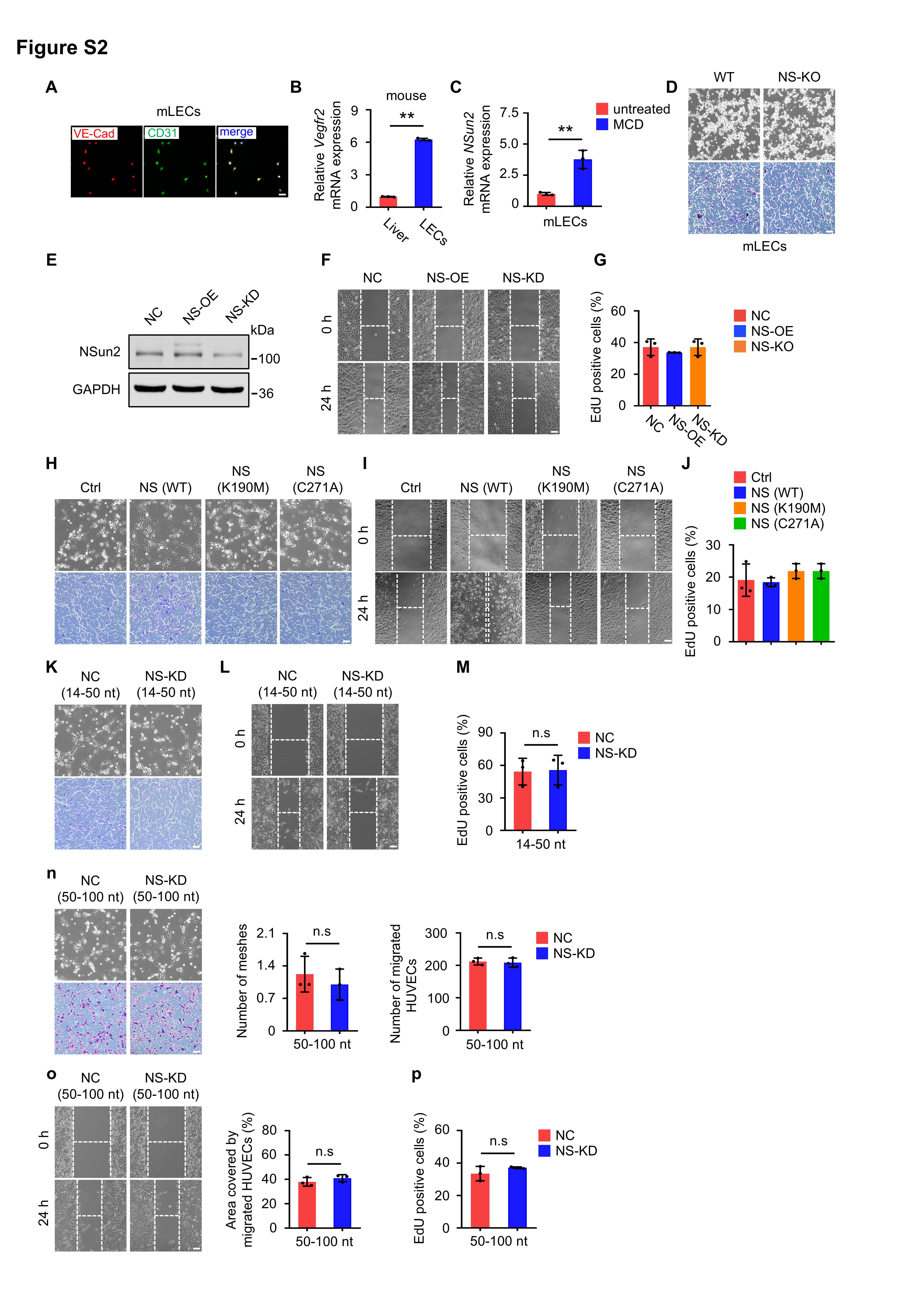


**Figure S2. *NSun2* deficiency inhibits endothelial cell migration.**

(A) Immunofluorescence staining was performed to detect the expression of vascular endothelial-cadherin (VE-cad) and CD31 in the mLECs. VE-cad, an endothelial cell marker. Scale bar: 100 μm. (B) The mRNA levels of *Vegfr2* in the liver and mLECs are shown. *Vegfr2* is a marker of mLECs. (C) The mRNA levels of *NSun2* in the mLECs of WT mice and NS-KD mice after MCD feeding. (D) Tube formation assay and cell transwell assay were performed to investigate the effect of *NSun2* expression on mLECs. Scale bar: 100 μm; n = 3 replicates. (E) *NSun2* knockdown or overexpression was performed to verify the effect of NSun2 by western blotting. (F) The effect of *NSun2* expression on the migratory ability of HUVECs. Scale bar: 100 μm; n = 3 replicates. (G) The effect of *NSun2* expression on the proliferation of HUVECs. n = 3 replicates. (H-J) The effect of rescuing *NSun2*-knockdown HUVECs with enzymatic-dead NSun2 on tube formation, cell transwell (H), wound healing (I) and EdU assays (J). Scale bar: 100 μm; n = 3 replicates. (K, L) Tube formation assay, cell transwell assay (K), and wound healing assay (L) were performed to investigate the effect of 14-50 nt small RNA on HUVECs. Scale bar: 100 μm; n = 3 replicates. (M) EdU assay showing the proliferation of isolated fragments (14-50 nt). n = 3 replicates. (N-P) Tube formation, cell transwell (N), wound healing (O) and EdU assays (P) were performed to investigate the effect of 14-50 nt small RNAs on HUVECs. n = 3 replicates. All data are presented as mean ± SD. **p* < 0.05, ***p* < 0.01, Student’s *t*-test.


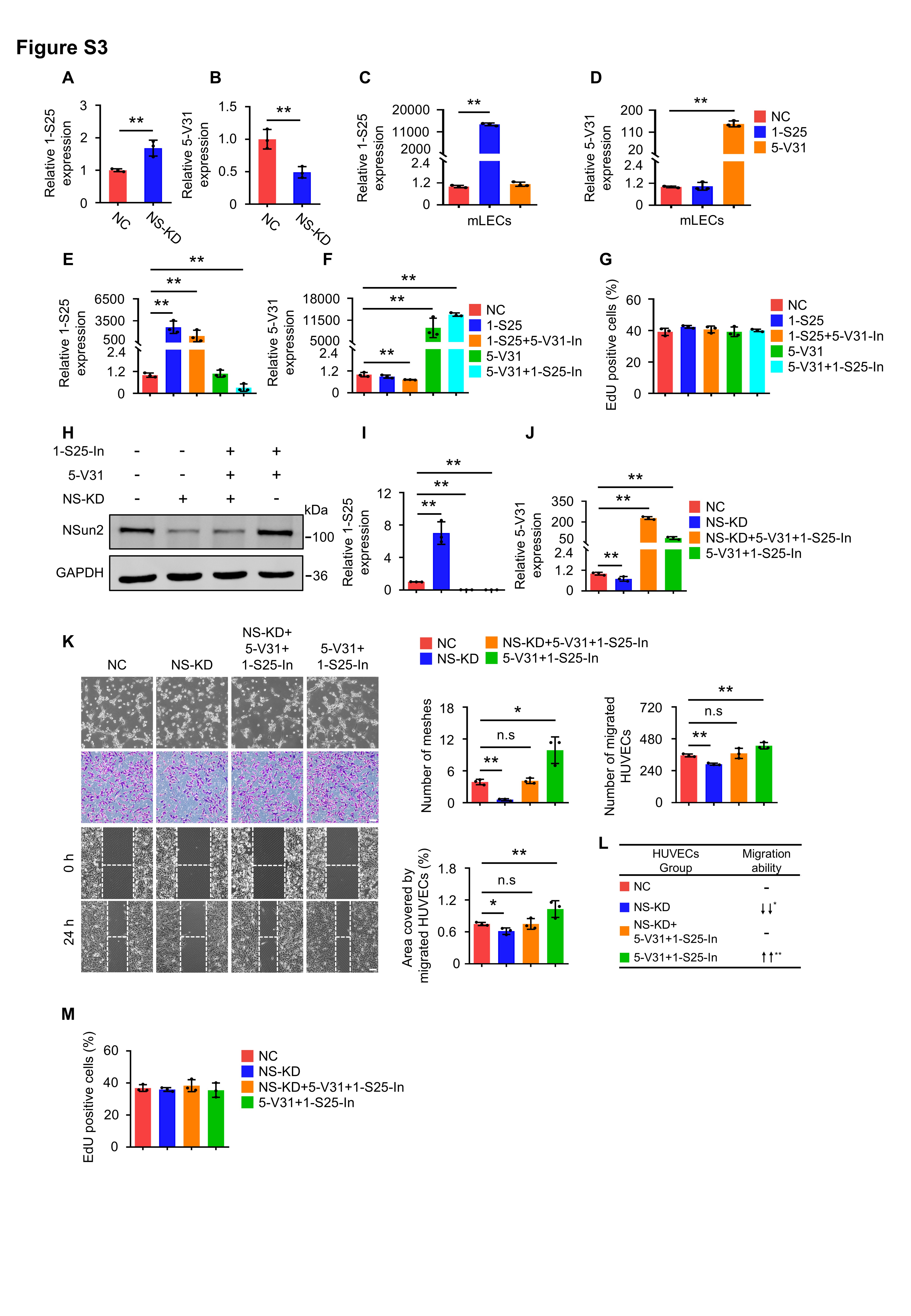


**Figure S3. The two tsRNAs influencing cell migration.**

(A, B) The expression levels of 1-S25 (A) and 5-V31 (B) tsRNAs in NS-KD HUVECs. (C, D) The levels of 1-S25 (C) and 5-V31 (D) were determined in each experimental condition. (E, F) The levels of 1-S25 (E) and 5-V31 (F) were determined in each experimental condition. (G) EdU assay showing the proliferation of 1-S25, 5-V31, and their combination. n = 3 replicates. (H-J) *NSun2*-knockdown HUVECs were rescued by introducing 5-V31 + 1-S25-In small RNA. The protein (H) level of NSun2 was determined in each condition. Expression of 1-S25 (I), and 5-V31 (J) were also determined. (K) *NSun2*-knockdown HUVECs were rescued by introducing 5-V31 + 1-S25-In small RNA. The tube formation assay, cell transwell assay, and wound healing assay were conducted to evaluate the effects of this rescue on the functional capabilities of the HUVECs. The results of these assays were analyzed statistically, and both quantitative data are presented to illustrate the outcomes. n = 3 replicates. (L) The chart illustrates the effect of 5-V31 with 1-S25-In small RNA groups on the cell migratory ability in NS-KD. An upward arrow indicates promotion of cell migratory ability, while a downward arrow indicates inhibition of cell migratory ability. (M) EdU assay of *NSun2*-knockdown HUVECs rescued by 5-V31 + 1-S25-In small RNA. n = 3 replicates. All data are presented as mean ± SD. **p* < 0.05, ***p* < 0.01, Student’s *t*-test.


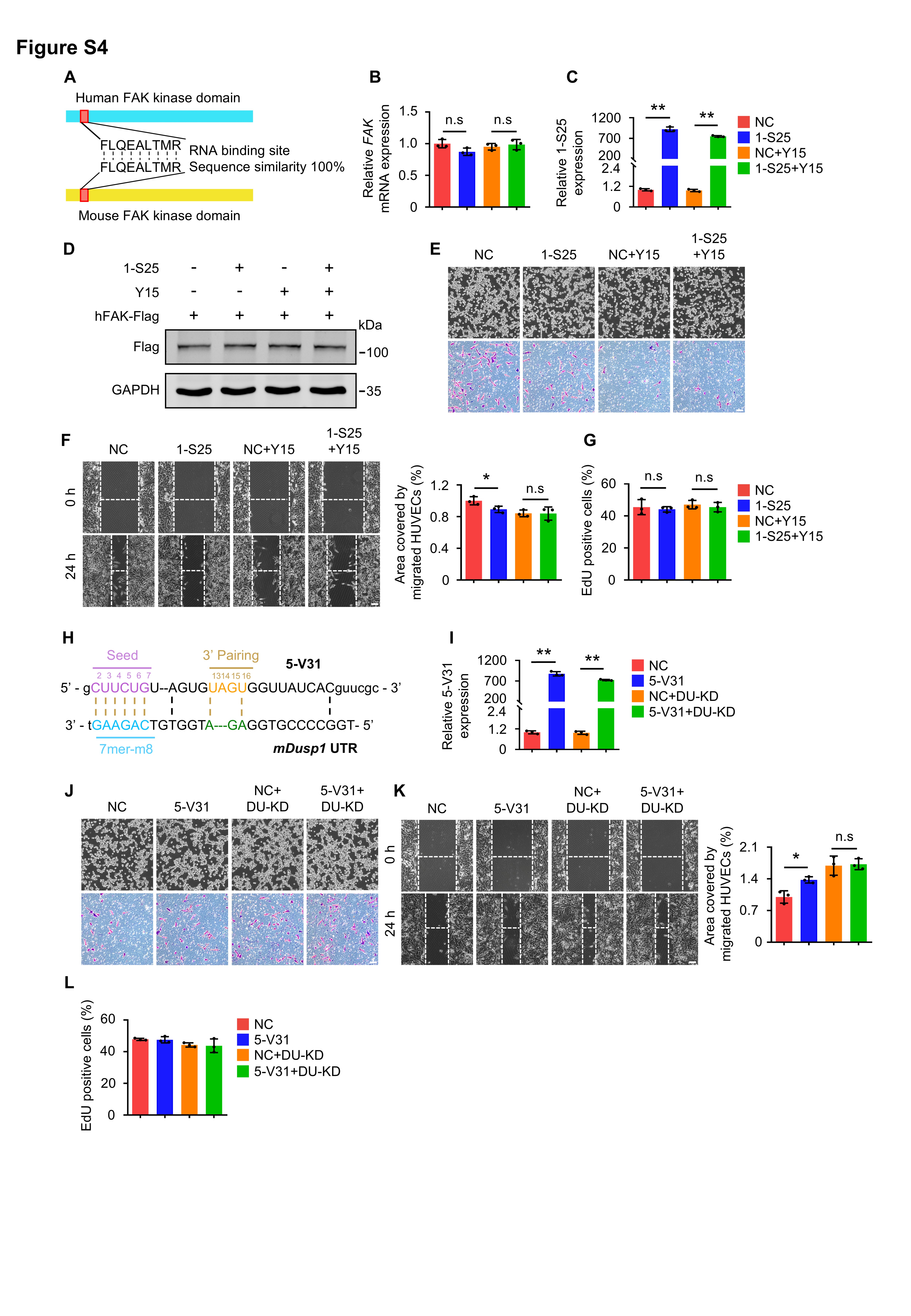


**Figure S4. The influence of 1-S25 and 5-V31 tsRNAs on the regulation of cell migration.**

(A) Protein sequences depicting the binding site of 1-S25 on human and mouse FAK protein. (B, C) The functional role of 1-S25 was validated by inhibiting FAK phosphorylation. The mRNA expression levels of *FAK* (B) and 1-S25 (C) in HUVECs were determined in each experimental condition. (D) Western blot analysis of Flag in HUVECs transfected with FAK-Flag. (E) Tube formation assay and cell transwell assay were performed in 1-S25-transfected HUVECs in which FAK was inhibited. n = 3 replicates. (F, G) Wound healing assay (F) and cell proliferation assay (G) were performed in 1-S25-transfected HUVECs in which FAK was inhibited. Scale bar: 100 μm; n = 3 replicates. (H) Predicted 5-V31-binding site at the 3′UTR of mouse *Dusp1* mRNA. (I) The relative expression level of 5-V31 in HUVECs was determined in each experimental condition. (J) Tube formation assay and cell transwell assay were performed in 5-V31-transfected HUVECs in which *DUSP1* was knocked down. Scale bar: 100 μm; n = 3 replicates. (K, L) Wound healing assay (K) and cell proliferation assay (L) were performed in 5-V31-transfected HUVECs in which *DUSP1* was knocked down. n = 3 replicates. All data are presented as mean ± SD. **p* < 0.05, ***p* < 0.01; Student’s *t*-test.


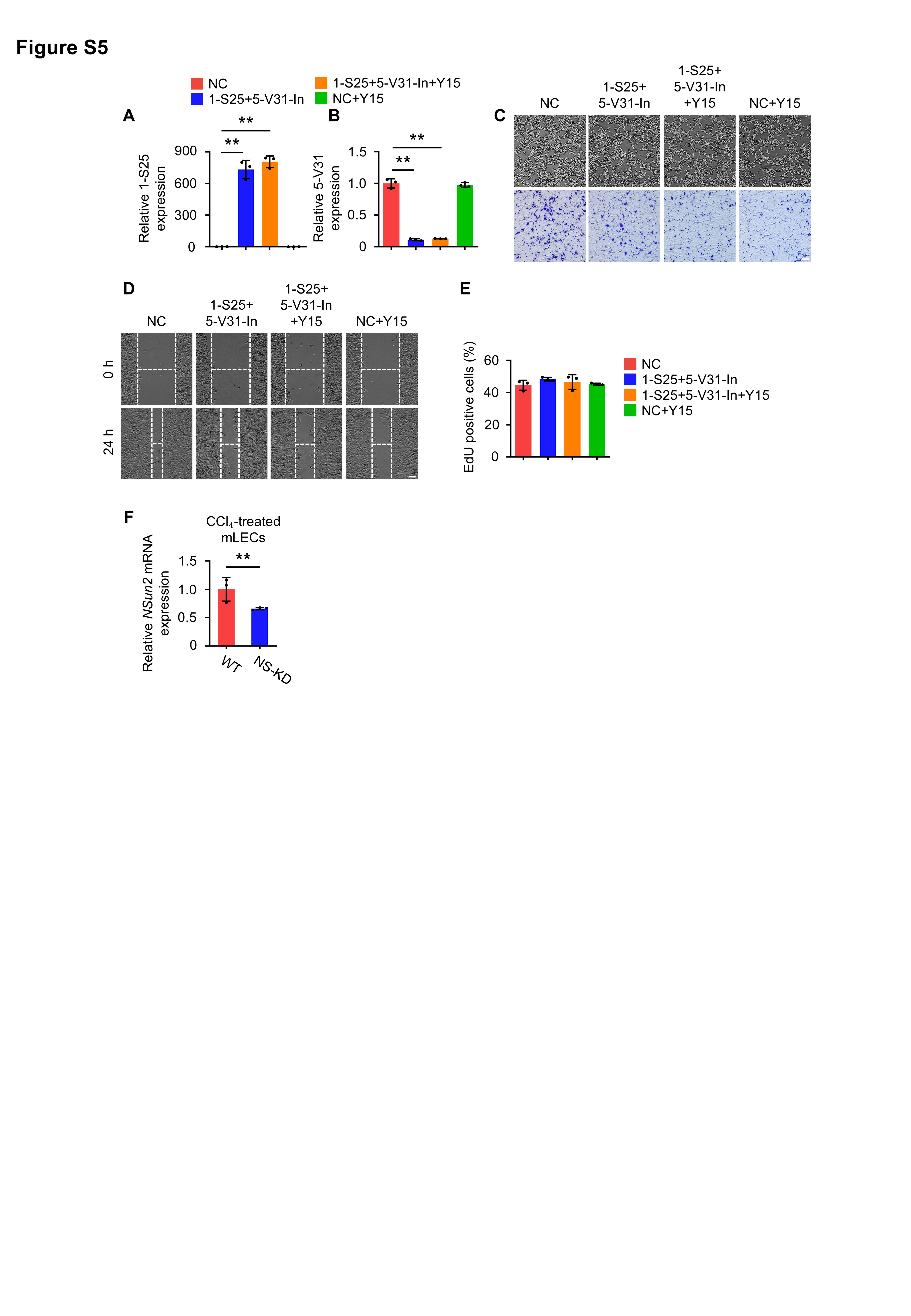


**Figure S5. 1-S25 and 5-V31 collaboratively target FAK to affect cell migration.**

(A, B) The expression levels of 1-S25 (A) and 5-V31 (B) in HUVECs were determined in each experimental condition. (C) Tube formation assay and cell transwell assay of FAK was performed in 1-S25- and 5-V31-In-transfected HUVECs in which FAK was inhibited. Scale bar: 100 μm; n = 3 replicates. (D, E) Wound healing assay (D) and cell proliferation assay (E) were performed. n = 3 replicates. (F) The expression level of NSun2 in mLECs under CCl_4_ treatment was determined by quantitative PCR. All data are presented as mean ± SD. **p* < 0.05, ***p* < 0.01, Student’s *t*-test.


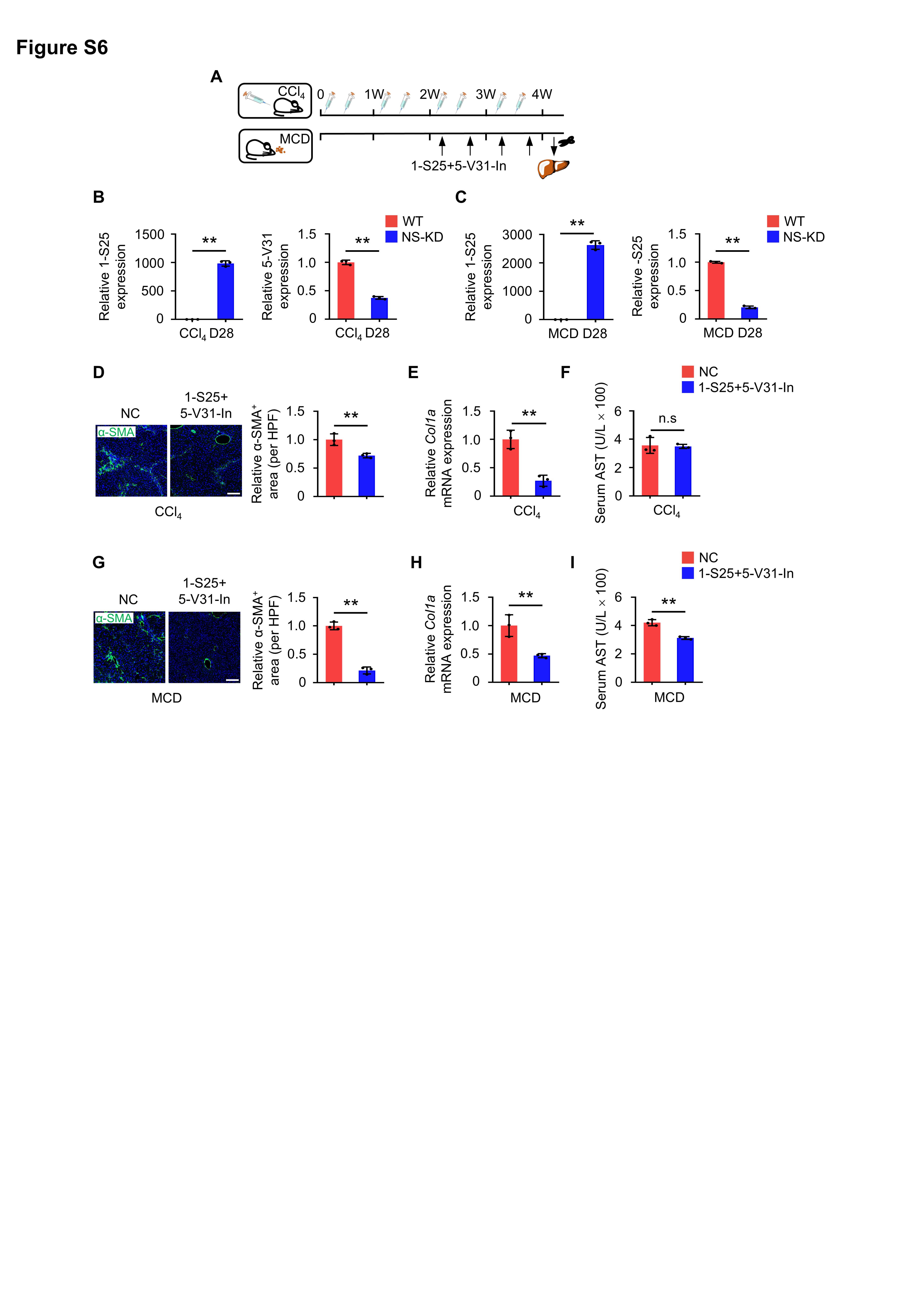


**Figure S6. The two tsRNAs alleviate liver fibrosis in the mouse model.**

(A) Schematic diagram illustrating the induction of liver fibrosis in the mouse models. NC small RNA or 1-S25 + 5-V31-In were injected twice a week during the last two weeks. (B, C) The expression levels of 1-S25 and 5-V31 in the liver of CCl_4_-induced (B) and MCD-induced (C) liver injury models were measured after injection with NC small RNA or 1-S25 + 5-V31-In. (D) Immunofluorescence staining was performed to detect the expression of α-SMA in the liver of mice with CCl_4_-induced liver fibrosis. Scale bar: 100 μm; n = 3 mice. (E) The mRNA levels of *Col1a* in the liver of CCl_4_-treated mice. (F) Mouse serum AST concentrations were assessed to determine the degree of CCl_4_-induced liver injury. n = 3 mice. (G) Immunofluorescence staining was performed to detect the expression of α-SMA in the livers of MCD-fed mice. Scale bar: 100 μm; n = 3 mice. (H) The mRNA levels of *Col1a* in the livers of MCD-fed mice. (I) Mouse serum AST concentrations were assessed to determine the degree of MCD-induced liver injury. n = 3 mice. All data are presented as mean ± SD. **p* < 0.05, ***p* < 0.01, Student’s *t*-test.

**Supplementary Materials and Methods**

**1 Ethical compliance**

The animal experiments carried out in this study were performed in full compliance with the guidelines and procedural standards set forth by the local Experimental Animal Ethics Committee and the Institutional Animal Care and Use Committee (IACUC).

**2 RNA extraction and qPCR**

For RNA extraction, 1 mg of tissue or 1 × 10^6^ cells per group was lysed with 1 mL of TRIzol reagent (Invitrogen, 15596018). To the lysate, 200 μL of chloroform was added, followed by centrifugation at 12,000 × g for 20 min at 4°C. Then, 400 μL of the upper aqueous phase was collected, and an equal volume of 70% ethanol was added. RNA extraction was then performed using the PureLink RNA Mini Kit (Invitrogen, 12183020). Subsequently, RNA was reverse-transcribed using M-MLV Reverse Transcriptase (Promega, M1705) to obtain cDNA. Reverse transcription for 1-S25 and 5-V31 was carried out using specific reverse transcription primers. qRT-PCR (quantitative real-time polymerase chain reaction) was performed using the Power SYBR Green PCR Master Mix (ABI, 4367659) reagent. GAPDH or U6 was used as the internal reference for normalization. Relative gene expression was analyzed based on the 2^(-ΔΔCt) method. The thermal cycle conditions for PCR amplification were as follows: initial denaturation at 95°C for 1 min, followed by 45 cycles of denaturation at 95°C for 15 s, annealing at 60°C for 15 s, and extension at 72°C for 45 s. Melting curve analysis was performed with a denaturation step at 95°C for 1 min, annealing at 60°C for 30 s, and subsequent denaturation at 95°C for 30 s. All reactions were conducted using AGILENT MX3005P equipment, and at least three replicates were used for each sample to ensure accuracy of the experimental results.

**3 Histopathologic examination**

Liver tissues were fixed in 4% paraformaldehyde (PFA) for over 24 hours. Following fixation, the tissues were dehydrated using a series of graded ethanol solutions and then embedded in paraffin. The solidified paraffin blocks were cut into 5 μm-thick sections for subsequent staining. For hematoxylin and eosin (H&E) staining (Solarbio, G1120-3), the tissue sections were rehydrated using a series of graded ethanol solutions. Subsequently, the sections were stained with hematoxylin to visualize cell nuclei and eosin to stain the cytoplasm. After staining, the sections were dehydrated and sealed with neutral gum for preservation. Sirius Red staining is commonly used to assess collagen deposition and fibrosis. For Sirius Red staining, the tissue sections were rehydrated and immersed in Sirius Red (Solarbio, G3632) staining solution for 5 mins. After staining, the sections were dehydrated and sealed with neutral gum. The stained sections were evaluated under a microscope to examine the tissue structure and assess the staining results. Data were collected and analyzed to gain insights into the characteristics of the liver tissue and any observed changes.

**4 Examination of liver function**

To assess the liver function of the mice, mice were fasted overnight. Subsequently, 800 μL of blood was collected from the fundus vein. The collected blood was allowed to stand at 4°C for 30 mins. Afterward, the blood was centrifuged at 3,000 × g for 15 mins and the supernatant serum was carefully collected. The obtained serum samples were subjected to liver function tests to measure the levels of alanine aminotransferase (ALT) and aspartate aminotransferase (AST). These measurements were conducted using an automated biochemical analyzer (Chemray 240) following standard protocols specific to the equipment. To ensure the accuracy and reliability of the results, each set of samples was analyzed in at least three replicates.

**5 Transcriptome sequencing and analysis**

RNA was extracted from the livers of mice with liver fibrosis and those of control mice, as well as from HUVECs transfected with synthetic 1-S25, 5-V31, or Ctrl. The purity and integrity of the RNA samples were evaluated using a NanoPhotometer spectrophotometer (IMPLEN, CA, USA) for assessing the RNA concentration and an Agilent 2100 bioanalyzer (Agilent Technologies, CA, USA) was used for evaluating RNA integrity. To generate the sequencing library, 1 μg of RNA was used, following the instructions provided in the NEBNext® UltraTM RNA Library Prep Kit for Illumina® (NEB, USA) manual. mRNAs were enriched using poly-T oligo-attached magnetic beads and subsequently randomly fragmented using NEB Fragmentation Buffer. The first strand of the mRNA transcript was synthesized using M-MuLV Reverse Transcriptase, followed by RNA degradation using RNase H. The second strand was synthesized using DNA polymerase I and RNase H. The resulting double-stranded cDNAs were subjected to end repair, A-tailing, and adaptor addition. cDNAs ranging from 250 to 300 bp in size were selectively purified using the AMPure XP system (Beckman Coulter, Beverly, USA) and their library quality was assessed using the AMPure XP system and Agilent Bioanalyzer 2100 system. The indexed samples were clustered using the cBot Cluster Generation System and the TruSeq PE Cluster Kit v3-cBot-HS (Illumina). Subsequently, sequencing was performed on an Illumina Novaseq platform, generating 150 bp paired-end reads.

The Illumina sequenced reads in FASTQ files were aligned to the GRCm38 genome for mice and GRCm38 genome for humans using STAR (v.2.5.3a), based on the species of the sequenced sample. The resulting BAM files were used for quantifying gene expression with StringTie (v2.1.4). Differential expression analysis was conducted using DESeq2 (1.30.1), considering genes that met the specified threshold of |Foldchange| >= 1 and p-values < 0.05 between the two comparison groups. Genes meeting these criteria were used for downstream analysis. Functional ontology enrichment and KEGG pathway enrichment analysis were performed using ClusterProfiler (4.6.2).

**6 RNA synthesis**

1-S25, 5-V31, 1-S25-In, 5-V31-In, NC, *NSun2* siRNA, *DUSP1* siRNA, and their corresponding siRNA-NC were synthesized by the TIAN YI HUI YUAN company. To enhance the efficacy of 1-S25, 5-V31-In, *NSun2* siRNA, and their control RNAs for *in vivo* applications, 2'-OMe modification and PS modification were incorporated at the 5' and 3' ends of the RNA molecules, spanning three bases. These modifications were introduced to improve cellular uptake and prevent degradation[1].

**7 Preparing RNA mixture for mouse injection**

The RNA (1-S25, 5-V31-In, *NSun2* siRNA, and their corresponding control RNA) were diluted to a concentration of 1 μg/μL in RNase-free sterile water. Concurrently, a 10% glucose solution (w/v) was prepared. Subsequently, the RNA solution, 10% glucose solution, *in vivo* transfection reagent (Entranster in Vivo, 18668-11-1), and RNase-free sterile water were gently mixed in a ratio of 2:4:1:1. The mixture was allowed to incubate at room temperature for 15 mins. The resulting reaction solution was then injected into the mice via the tail vein at a dose of 10 μL/g.

**8 Culturing cells and transfection**

The HUVEC cell line was purchased from Procell, while LSECs and LPCs were isolated from mouse liver. HUVECs were cultured in human vein endothelial cell culture medium (OKA, F12684), LSECs were cultured in mouse liver sinusoidal endothelial medium, and LPCs were cultured in mouse liver parenchymal cell culture medium. The culture medium was replaced daily, and cells were passaged as needed using 0.25% trypsin containing EDTA. Authentication of HUVECs was performed by Short Tandem Repeat (STR) analysis. For cell transfection experiments, Lipofectamine 3000 (Thermo, L3000015) reagent was used in accordance with the manufacturer's instructions. Transfection experiments included *NSun2* siRNA, *DUSP1* siRNA, control siRNA, 50-100 nt RNA, 14-50 nt RNA, 1-S25, 5-V31, 1-S25-In, 5-V31-In, NC, as well as plasmid transfection. To investigate the relationship between 1-S25 and FAK phosphorylation, the culture medium was supplemented with 10 μM Y15 (InvivoChem, V3389-500mg) to inhibit FAK phosphorylation. To confirm the effect of NSun2 alterations in mLECs on FAK phosphorylation in fibrotic mice, mLECs were treated with 200 nM CCl_4_ for 12 hours, and the cells were collected for subsequent analysis.

**9 Plasmid vector construction**

The human *NSun2* coding sequence (CDS) sequence (NM_017755.6) was amplified from 293T cells using Tks Gflex DNA polymerase (Takara, R060A). Point-mutation primers were used to generate a nonsense sequence corresponding to the *NSun2* siRNA target position. In addition, two mutant sequences of *NSun2*, C271A and K190M, were also generated. These sequences were then ligated into the pCDH (SBI, CD521A-1) plasmid vector containing the EF1α promoter for subsequent experiments. To study the function of the *FAK* gene, the CDS sequence of human *FAK* was synthesized by Beijing Tsingke Biotech Co., Ltd. The *FAK* E471Q mutant sequence was created using point mutation primers based on protein sequencing results. These sequences were inserted into the pCDH plasmid containing the EF1α promoter as the vector for further experiments. To verify the binding ability of 5-V31 and *DUSP1*, the predicted binding sequence of *DUSP1* was artificially linked to a dual-luciferase plasmid (pmirGLO, MiaoLingBio, P0198) for dual-luciferase reporter experiments.

**10 Cell proliferation assay**

To assess cell proliferation ability, we performed a 5-ethynyldeoxyuridine (EdU) incorporation assay. After 48 hours of transfection, HUVECs were digested and resuspended, and then plated in a 96-well cell culture plate at a density of 4 × 10^3^ cells per well. Following cell attachment, the cells were treated with 50 μM EdU for 2 hours. Subsequently, the cells were fixed with 4% PFA for 1 hour. To neutralize the PFA, 2 mg/mL glycine (Sigma, G8790-100G) was applied for 5 mins, followed by three washes with PBS. The Cell-Light Apollo 567 staining kit (RiboBio, C10310-1) was used according to the manufacturer's instructions to stain the cells, in a dark environment. Nuclei were counterstained with Hoechst 33342. The results were observed using an inverted fluorescence microscope (LEICA, DM2500), and three random fields of view were captured for each well to ensure accuracy.

**11 tsRNA sequencing and analysis**

Small RNAs were isolated from HUVECs to construct a small RNA library, ensuring a minimum of two biological replicates to ensure reliable results. Following assessment of RNA quality, the RNA samples underwent modification and purification steps in preparation for subsequent experiments. The NEBNext Multiplex Small RNA Library Prep Set for Illumina (E7300L) was employed for the preparation of tsRNA sequencing libraries. This involved various steps including 5'-adapter ligation, 3'-adapter ligation, cDNA synthesis, PCR amplification, and selection of amplicons ranging from 134 to 160 base pairs through PCR to enhance product yield. The concentration and quality of the resulting sequencing libraries were evaluated using the Agilent 2100 Bioanalyzer. Based on the assessment, the libraries were pooled in equal proportions. The library DNA was linearized using 0.1 M NaOH, and sequencing was performed using the NextSeq 500/550 V2 Kit (Illumina, #FC-404-2005) according to the instructions provided by the NextSeq system (Illumina NextSeq 500), with a sequencing cycle of 50. All procedures were carried out by Aksomics Corporation in accordance with the agreed-upon protocol.

Initially, raw data files in FASTQ format were generated by the Illumina sequencer, and the sequencing quality was evaluated using FastQC. Clean reads were obtained by applying quality filtering and real-time base calling (Solexa pipeline version 1.8, Off-Line Base Caller software, version 1.8) to the raw sequence data from the Illumina NextSeq. The resulting clean reads were saved in FASTQ format. Further quality control was performed using Illumina tools, and the sequencing reads underwent 5'- and 3'-adapter trimming based on the clean reads. Reads that did not meet the length criteria (length < 14 nt or length > 40 nt) were discarded and recorded as trimmed reads in FASTA format, using cutadapt. Trimmed reads that passed Illumina quality control were aligned to mature tRNA sequences, allowing for one mismatch, followed by alignment of non-mapped reads to precursor tRNA sequences, also allowing for one mismatch, using bowtie software. Expression profiles of tsRNAs and miRNAs were calculated based on the counts of mapped reads, which were then normalized to counts per million total aligned reads (CPM). Differentially expressed tsRNAs were identified using the R package edgeR, with count values used for screening. A heat map depicting expressed tsRNAs, highlighting differential expression between the experimental and control groups, was generated using the pheatmap package in R.

**12 Small RNA target prediction**

To investigate the functional roles of small RNAs, we performed transcriptome target prediction for 1-S25 and 5-V31. For this analysis, we integrated two algorithms: miRanda and TargetScan. miRanda utilizes a dynamic programming algorithm that analyzes RNA secondary structure and free energy to identify potential binding sites. This enables the detection of binding sites of various seed sequences and enhances its ability to discover target interactions. TargetScan utilizes mRNA and miRNA expression profiles to identify the characteristics of site sequences with biological significance. It employs relatively conservative scoring models and can search for specific binding motifs, such as 8mer, 7mer-m8, 7mer-1a, 2-7, and 2-8 nucleotide matches.

**13 Proteome detection and analysis**

For further protein analysis, proteome sequencing was performed by Novogene Co. Ltd. First, the supernatant was freeze-dried to obtain a dry powder, which was then dissolved in lysis buffer (8 M urea, 100 mM TEAB, pH 8.5). The solution underwent reduction with 10 mM DTT at 56°C for 1 hour, followed by alkylation using iodoacetamide for 1 hour at room temperature in the dark. Protein concentration was determined using the Bradford protein quantification kit, and 20 μg of each sample was loaded onto a 12% SDS-PAGE gel for assessment of protein amount. After confirming the sample quality, 120 μg of each protein sample was taken and mixed with DB lysis buffer, trypsin, and 100 mM TEAB buffer. The mixture was digested at 37°C for 4 hours, followed by digestion with trypsin and CaCl_2_ overnight. Formic acid was added to lower pH, and centrifugation was performed. The supernatant was loaded onto a C18 desalting column, washed with washing buffer, and eluted with elution buffer. The eluate of each sample was collected and lyophilized. The lyophilized powder was dissolved in solution A, and centrifuged. Next, 1 μg of the supernatant was subjected to peptide separation on a self-made C18 Nano-Trap column. The separated peptides were then analyzed using a Q Exactive series mass spectrometer (Thermo Fisher) to obtain raw data for further analysis.

The spectral results were searched through a protein database using Proteome Discoverer 2.4 software (PD 2.4, Thermo). The software parameters used for searching included a mass tolerance of 10 ppm for precursor ions and 0.02 Da for productions. Fixed modifications included carbamidomethylation, while oxidation of methionine (M) was considered as a dynamic modification. N-terminal modifications such as acetylation, Met-loss, and Met-loss + acetylation were also taken into account. Up to 2 missed cleavage sites were allowed during searching. The results were then filtered using specific criteria. Only Peptide Spectrum Matches (PSMs) with a credibility over 99% and proteins identified with at least one unique peptide were considered to be used for further analysis. False discovery rate (FDR) verification was performed, and any result with FDR greater than 1% was discarded. Furthermore, *t*-tests were performed to assess the statistical significance of the identified proteins in order to determine the differentially expressed proteins between the experimental and control groups. In addition, a protein 3D structure diagram of FAK was created with ChimeraX.

**14 Tyrosinase activity assay**

To assess the effect of 1-S25 on FAK tyrosinase activity, we performed a tyrosinase activity assay. HUVECs were co-transfected with FAK-flag, control, and 1-S25. After 48 hours, approximately 1 × 10^6^ cells were collected. The cells were lysed on ice for 20 mins, and the supernatant was obtained by centrifugating at 12,000 × g for 5 mins. The supernatant was then incubated with equilibrated FLAG antibody agarose beads (Abbkine, ABT2013) overnight at 4°C with rotation. After incubation, the agarose beads were collected by centrifugation at 3,000 × g for 5 mins to obtain the protein. To measure tyrosinase activity of the purified FAK protein, we used the Tyrosinase Activity Assay Kit (BC4055) following the manual.

**15 Dual luciferase assay**

To investigate the impact of 5-V31 on the target sequence, a dual luciferase assay was conducted. HUVECs were transfected with a luciferase plasmid containing the predicted target sequence of 5-V31, along with a control plasmid. The Firefly & Renilla Dual Luciferase Assay Kit (X-Y Bio, XY9F6075) was used for this assay, following the provided instructions. This assay utilizes two distinct luciferases: Firefly luciferase and Renilla luciferase. Firefly luciferase is commonly used as a reporter gene, while Renilla luciferase served as an internal control.

**16 Data analysis for scRNA-seq and phosphoproteome**

scRNA-seq data with the GEO number GSE129516 was re-analyzed using the Seurat package (version 4.3.0). The expression and proportion of *NSun2* and *DUSP1* were compared and visualized between the healthy and NASH groups. Additionally, phosphoproteome data the study by Josiah Hardesty et al. were utilized to analyze the differential phosphorylation degree of specific genes between two groups: alcohol-associated cirrhosis (AC) vs the control and alcohol-associated hepatitis (AH) vs the control. All plots were generated using the R packages ggplot2 (version 3.4.2) and pheatmap (version 1.0.12).

**References**

1. Ying S, Li P, Wang J *et al.* tRF-Gln-CTG-026 ameliorates liver injury by alleviating global protein synthesis. *Signal Transduct Target Ther*. 2023; **8**(1): 144. doi: 10.1038/s41392-023-01351-5

**Table S1.** **Markedly altered tRF-1 and tRF-5 small RNAs.**

| tRF-ID | sequences |  |
| --- | --- | --- |
| 1-G17 | GTGTCAGTTTTTCTCTT | tRF1 log_2_FC > 0.5 *p* < 0.05  compare WT with NS-KD |
| 1-G26 | GTGTCAGTTTTTCTCTTCAGCTTTTT |  |
| 1-V15 | CCTCCTTCTGCTTTT |  |
| 1-A14 | CCTCTTTGAGCTTT |  |
| 1-S16 | GAAGCGGGTGCTCTTT |  |
| 1-G19 | TTCAAAGGTGAACGTTTTA |  |
| 1-S25 | GCTAAGGAAGTCCTGTGCTCAGTTT |  |
| 1-L16 | AAGAGGAGTTGTTTTT |  |
| 1-S19 | GCTGCATAGCAAGCCTTTT |  |
| 1-S25 | GCTAAGGAAGTCCTGTGCTCAGTTTT |  |
| 1-A17 | ATAGGTATTAAGGTTTT |  |
| 1-G16 | TGCGGTACCACTTTTG |  |
| 1-G17 | TTCAAAGGTGAACGTTT |  |
| 1-G15 | TGCGGTACCACTTTT |  |
| 1-S14 | TTTCTGGAGGTTTT |  |
| 5-H28 | GCCGTGATCGTATAGTGGTTAGTACTCT | tRF5 log_2_FC < 0.5 *p* < 0.05  compare WT with NS-KD |
| 5-G43 | GACTCTGAATCCAG |  |
| 5-H29 | GCCGTGATCGTATAGTGGTTAGTACTCTG |  |
| 5-V31 | GCTTCTGTAGTGTAGTGGTTATCACGTTCGC |  |
| 5-G30 | TCCCATATGGTCTAGCGGTTAGGATTCCTG |  |
| 5-S16 | GTCACGGTGGCCGAGT |  |
| 5-G28 | TGGACTCTGAATCC |  |
| 5-G16 | GCATTGGTGGTTCAGT |  |
| 5-P33 | GGCTCGTTGGTCTAGTGGTATGATTCTCGCTTT |  |
| 5-SG16 | GACGAGGTGGCCGAGT |  |
| 5-T58 | ATTCCGGCTCGAAGGACCA |  |
| 5-G15 | TCCCTGGTGGTCTAG |  |
| 5-H30 | GCCGTGATCGTATAGTGGTTAGTACTCTGC |  |
| 5-L16-1 | GTCAGGATGGCCGAGT |  |
| 5-L16-6 | GTCAGGATGGCCGAGC |  |

**Table S2. Predicted target genes of tRF-1-S25.**

| **1-S25 predicted target genes** | | | | |
| --- | --- | --- | --- | --- |
| **Gene** | **Context+** | **Structure** | **Energy** | **UTR Length** |
| TIPIN | -0.42 | 144 | -13.72 | 768 |
| MUC7 | -0.33 | 170 | -25.11 | 1118 |
| PABPC5 | -0.391 | 145 | -12.99 | 1858 |
| GIMAP4 | -0.329 | 150 | -17.01 | 1700 |
| ARG1 | -0.385 | 174 | -22.98 | 417 |
| GABARAPL2 | -0.402 | 147 | -19.17 | 512 |
| VPS37D | -0.321 | 140 | -14.05 | 734 |
| CATG00000007173.1 | -0.328 | 142 | -12.58 | 1000 |
| ARL14EPL | -0.326 | 140 | -12.58 | 1171 |
| SH3YL1 | -0.422 | 151 | -14.88 | 653 |
| PGM2 | -0.355 | 147 | -13.73 | 2277 |
| DGKK | -0.321 | 151 | -15.72 | 3531 |
| CFAP44 | -0.333 | 140 | -14.67 | 4627 |
| CATG00000094142.1 | -0.399 | 149 | -13.22 | 1000 |
| OR2T29 | -0.36 | 150 | -13.73 | 1632 |
| ZBTB3 | -0.303 | 144 | -13.74 | 3770 |
| IL36B | -0.363 | 142 | -12.94 | 583 |
| GGA2 | -0.454 | 149 | -15.73 | 5408 |
| CRTAM | -0.309 | 153 | -13.73 | 1240 |
| ZNF550 | -0.342 | 155 | -19.04 | 2466 |
| CATG00000091028.1 | -0.347 | 158 | -23.61 | 1000 |
| ZNF350 | -0.3 | 150 | -15.25 | 971 |
| CATG00000043785.1 | -0.356 | 160 | -20.16 | 1000 |
| TMEM260 | -0.335 | 146 | -14.04 | 2018 |
| LRRC27 | -0.311 | 150 | -19.28 | 4676 |
| SNX31 | -0.309 | 141 | -13.3 | 981 |
| MSH4 | -0.414 | 140 | -12.27 | 351 |
| ASPHD2 | -0.391 | 156 | -15.86 | 1809 |
| RMDN3 | -0.341 | 146 | -23.04 | 658 |
| MAX | -0.333 | 149 | -14.96 | 932 |
| DSC2 | -0.309 | 156 | -18.36 | 2039 |
| DOK6 | -0.338 | 188 | -36.56 | 7704 |
| POLI | -0.322 | 145 | -13.58 | 3785 |
| RBL2 | -0.362 | 144 | -15.53 | 1349 |
| AC117834.1 | -0.333 | 145 | -14.08 | 755 |
| CECR2 | -0.315 | 155 | -21.55 | 492 |
| CDH8 | -0.346 | 158 | -23.25 | 6366 |
| AC009690.1 | -0.359 | 153 | -14.51 | 1644 |
| LAT | -0.319 | 144 | -14.52 | 602 |
| DNAH11 | -0.385 | 146 | -13.81 | 585 |
| SLC30A9 | -0.387 | 144 | -17.7 | 4331 |
| UBE2C | -0.368 | 146 | -14.42 | 190 |
| FOXM1 | -0.313 | 151 | -16.06 | 960 |
| SLC17A2 | -0.322 | 146 | -14.29 | 563 |
| WASHC5 | -0.381 | 157 | -20.04 | 357 |
| HSD17B4 | -0.393 | 150 | -15.68 | 338 |
| HNRNPUL2 | -0.31 | 154 | -17.96 | 2674 |
| CD164 | -0.333 | 151 | -17.08 | 2337 |
| CNIH1 | -0.383 | 143 | -14.09 | 4254 |
| SUN1 | -0.325 | 146 | -15.49 | 1571 |
| PRRC1 | -0.33 | 141 | -12.58 | 1203 |
| LIN52 | -0.334 | 159 | -16.84 | 2514 |
| VCPKMT | -0.324 | 155 | -16.19 | 1067 |
| WAPL | -0.396 | 153 | -17.25 | 2287 |
| LRRC19 | -0.355 | 157 | -20.9 | 2385 |
| OR1S1 | -0.355 | 150 | -14.3 | 362 |
| MED7 | -0.371 | 140 | -13.04 | 5449 |
| LINC00672 | -0.328 | 153 | -15.06 | 3088 |
| NXPH3 | -0.382 | 157 | -14.96 | 4509 |
| KCTD15 | -0.448 | 152 | -18.06 | 2813 |
| PTBP3 | -0.395 | 156 | -23.81 | 6135 |
| CHURC1 | -0.375 | 164 | -25.23 | 3138 |
| KRTAP4-12 | -0.39 | 157 | -15.28 | 426 |
| CATG00000007582.1 | -0.351 | 157 | -24.75 | 1000 |
| TMEM41A | -0.36 | 152 | -17.89 | 1955 |
| MED15 | -0.312 | 164 | -20.8 | 915 |
| RBM45 | -0.407 | 152 | -18.07 | 268 |
| TTC21B | -0.303 | 140 | -15.4 | 1432 |
| CATG00000053459.1 | -0.441 | 153 | -15 | 1000 |
| MPDU1 | -0.349 | 144 | -13.86 | 797 |
| CCDC34 | -0.369 | 154 | -18.96 | 323 |
| ACTR6 | -0.386 | 162 | -23.61 | 508 |
| CATG00000100722.1 | -0.4 | 140 | -12.58 | 1000 |
| MUC17 | -0.302 | 152 | -18.46 | 815 |
| GOLT1B | -0.324 | 149 | -17.67 | 2704 |
| FFAR2 | -0.347 | 146 | -14.11 | 1060 |
| PRL | -0.411 | 147 | -15.31 | 158 |
| PTGR2 | -0.436 | 140 | -12.58 | 2650 |
| DLGAP5 | -0.445 | 147 | -13.73 | 712 |
| RBL1 | -0.399 | 147 | -13.73 | 2680 |
| SPAAR | -0.337 | 141 | -20.07 | 995 |
| TTC39A | -0.397 | 152 | -17.36 | 899 |
| R3HCC1 | -0.384 | 157 | -20.69 | 1326 |
| EDN3 | -0.377 | 151 | -20.15 | 1533 |
| HEMK1 | -0.362 | 149 | -16.09 | 15751 |
| TBC1D4 | -0.353 | 155 | -18.26 | 3306 |
| KCNAB3 | -0.355 | 155 | -16.17 | 1244 |
| FAM110B | -0.417 | 144 | -12.58 | 2856 |
| GTF3C2 | -0.327 | 163 | -24.27 | 932 |
| BRWD1 | -0.345 | 168 | -19.01 | 2851 |
| PEX13 | -0.311 | 150 | -19.68 | 3794 |
| ENKUR | -0.369 | 149 | -13.81 | 2391 |
| SNCB | -0.318 | 175 | -30.82 | 727 |
| GRIK5 | -0.309 | 158 | -27.92 | 329 |
| TRABD | -0.397 | 146 | -20.37 | 463 |
| CENPF | -0.418 | 162 | -18.02 | 5155 |
| ZNFX1 | -0.352 | 149 | -14.37 | 1367 |
| MFAP3 | -0.406 | 147 | -16.65 | 1320 |
| CATG00000010356.1 | -0.347 | 164 | -26.21 | 1000 |
| LUM | -0.408 | 150 | -17.11 | 1536 |
| ZNF878 | -0.334 | 146 | -17.48 | 2814 |
| CATG00000039159.1 | -0.371 | 148 | -15.06 | 1000 |
| CNIH4 | -0.338 | 142 | -13.79 | 4150 |
| CATG00000015176.1 | -0.36 | 158 | -15.55 | 1000 |
| PRTG | -0.306 | 158 | -14.43 | 8471 |
| CATG00000072992.1 | -0.325 | 145 | -17.77 | 1000 |
| SNURF | -0.4 | 148 | -17.55 | 371 |
| WWOX | -0.371 | 149 | -13.73 | 871 |
| PRKG2 | -0.322 | 147 | -15.06 | 2338 |
| RNF141 | -0.393 | 147 | -13.73 | 3263 |
| PELO | -0.388 | 158 | -20.78 | 5222 |
| TOR1AIP1 | -0.377 | 162 | -16.44 | 1469 |
| CELF6 | -0.409 | 153 | -14.51 | 466 |
| OIP5 | -0.305 | 150 | -16.22 | 4761 |
| LRP11 | -0.413 | 155 | -15.06 | 1797 |
| MAPRE1 | -0.374 | 140 | -12.58 | 1677 |
| P2RX3 | -0.386 | 142 | -13.8 | 603 |
| DOCK5 | -0.321 | 162 | -21.97 | 4418 |
| MAGEB4 | -0.385 | 162 | -21.15 | 1015 |
| PELP1 | -0.356 | 146 | -12.58 | 1665 |
| TRPM3 | -0.399 | 140 | -12.58 | 6890 |
| FRZB | -0.339 | 156 | -21.03 | 1574 |
| HMOX2 | -0.411 | 142 | -15.72 | 974 |
| SHOX | -0.306 | 146 | -18.82 | 2187 |
| TDGF1 | -0.355 | 151 | -12.83 | 1213 |
| AQP4 | -0.356 | 167 | -24.87 | 4173 |
| DMRT1 | -0.394 | 147 | -13.86 | 951 |
| TSPO2 | -0.354 | 160 | -20.58 | 200 |
| SNX16 | -0.336 | 142 | -14.97 | 1910 |
| NIP7 | -0.384 | 144 | -12.58 | 1593 |
| MAVS | -0.353 | 156 | -17.59 | 9976 |
| COA5 | -0.306 | 150 | -16.47 | 4937 |
| DMGDH | -0.336 | 150 | -17.83 | 1117 |
| CATG00000007150.1 | -0.31 | 159 | -22.94 | 1000 |
| CTDNEP1 | -0.321 | 141 | -13.3 | 567 |
| VWA2 | -0.309 | 153 | -21.73 | 3263 |
| FOSL1 | -0.313 | 149 | -18.21 | 412 |
| PANX1 | -0.342 | 158 | -16.83 | 4738 |
| ZDHHC15 | -0.375 | 157 | -17.03 | 4551 |
| MBD5 | -0.378 | 166 | -20.13 | 5295 |
| PNLDC1 | -0.415 | 143 | -12.86 | 186 |
| UBE2F | -0.325 | 156 | -21.18 | 1444 |
| GABRG1 | -0.411 | 146 | -12.58 | 5219 |
| KLF5 | -0.345 | 157 | -17.92 | 1656 |
| CEP104 | -0.313 | 173 | -28.24 | 3408 |

**Table S3. Predicted target genes of tRF-5-V31.**

| **5-V31 predicted target genes** | | | | |
| --- | --- | --- | --- | --- |
| **Genes** | **Context+** | **Structure** | **Energy** | **UTR Length** |
| PSMC6 | -0.376 | 168 | -16.48 | 970 |
| AKAP7 | -0.305 | 166 | -22.77 | 1809 |
| EGLN1 | -0.316 | 149 | -19.84 | 3722 |
| COLEC10 | -0.324 | 158 | -25.02 | 9372 |
| BLOC1S1 | -0.303 | 162 | -19.21 | 786 |
| DMP1 | -0.325 | 141 | -19 | 1041 |
| ALG2 | -0.35 | 141 | -17.79 | 1509 |
| DUSP1 | -0.345 | 147 | -22.31 | 1875 |
| ZNF451 | -0.337 | 167 | -22.24 | 1838 |
| TBC1D26 | -0.33 | 144 | -21.34 | 866 |
| CALR | -0.352 | 152 | -18.81 | 1679 |
| FPR1 | -0.323 | 152 | -19.47 | 4810 |
| SNX9 | -0.306 | 165 | -21.81 | 2239 |
| CNTNAP5 | -0.336 | 166 | -21.05 | 6946 |
| ATG101 | -0.3 | 154 | -20.22 | 305 |
| EPDR1 | -0.316 | 162 | -21.63 | 1545 |
| CHD1 | -0.343 | 168 | -20.62 | 2395 |
| SNRNP27 | -0.31 | 153 | -17.32 | 1264 |
| CD300E | -0.303 | 143 | -17.49 | 2770 |
| ALOX15 | -0.326 | 157 | -20.23 | 2039 |
| CATG00000079193.1 | -0.315 | 179 | -21.83 | 1000 |
| LRTM2 | -0.336 | 160 | -19.52 | 2031 |
| EPHB2 | -0.335 | 162 | -23.17 | 1458 |
| BCL2L2-PABPN1 | -0.344 | 142 | -16.17 | 903 |
| UTP25 | -0.316 | 167 | -22.48 | 6118 |
| UFM1 | -0.331 | 169 | -24.91 | 347 |
| TMEM43 | -0.347 | 168 | -20.84 | 3377 |
| AIPL1 | -0.303 | 162 | -18.3 | 1723 |
| ZDHHC20 | -0.391 | 181 | -22.88 | 4527 |
| SOSTDC1 | -0.301 | 165 | -20.49 | 2258 |
| SPATA6 | -0.333 | 161 | -17.72 | 3341 |
| OR10T2 | -0.37 | 164 | -16.95 | 50 |
| WDR3 | -0.673 | 329 | -33.73 | 7095 |
| IKZF2 | -0.302 | 146 | -16.17 | 1997 |
| OR51D1 | -0.358 | 177 | -31.79 | 2295 |
| CATG00000039070.1 | -0.309 | 168 | -23.94 | 1000 |
| HLA-DPA1 | -0.319 | 162 | -19.77 | 791 |
| DDX55 | -0.31 | 147 | -22.82 | 1537 |
| H2AFV | -0.328 | 158 | -20.97 | 273 |
| ANKRD34C | -0.336 | 145 | -26.24 | 3347 |
| TMX1 | -0.392 | 159 | -17.2 | 4541 |
| POLI | -0.38 | 153 | -18.77 | 3785 |
| ODF3L1 | -0.307 | 162 | -24.24 | 148 |
| ADIPOR1 | -0.361 | 161 | -17.23 | 750 |
| LBR | -0.315 | 156 | -18.67 | 1800 |
| ZNF695 | -0.306 | 160 | -20.37 | 1644 |
| TUBGCP4 | -0.332 | 148 | -18.02 | 359 |
| PABPN1 | -0.344 | 142 | -16.17 | 903 |
| ALKBH6 | -0.314 | 157 | -20.18 | 446 |
| MAS1 | -0.326 | 158 | -17.03 | 143 |
| CYP2R1 | -0.335 | 159 | -21.79 | 682 |
| ARL5A | -0.361 | 158 | -17.29 | 7005 |
| CTDSPL2 | -0.335 | 156 | -17.8 | 4864 |
| BCAS1 | -0.349 | 161 | -17.25 | 1381 |
| ACAD10 | -0.323 | 145 | -16.02 | 621 |
| DCHS2 | -0.379 | 155 | -16.29 | 584 |
| AC009690.1 | -0.342 | 151 | -19.75 | 1644 |
| AC079210.1 | -0.321 | 145 | -16.17 | 2833 |
| RMND5A | -0.312 | 144 | -24.26 | 4637 |
| KLHL20 | -0.312 | 140 | -16.02 | 2285 |
| ZNF213 | -0.339 | 151 | -21.35 | 1458 |
| SLC9A1 | -0.312 | 157 | -21.49 | 1498 |
| VPS50 | -0.332 | 174 | -26.35 | 2687 |
| CLMP | -0.3 | 164 | -21.47 | 3696 |
| SLC17A8 | -0.334 | 140 | -15.69 | 1900 |
| RBM12 | -0.328 | 159 | -22.13 | 3599 |
| DLG3 | -0.319 | 151 | -18.13 | 3218 |
| RAB2A | -0.339 | 162 | -17.52 | 3774 |
| GJD3 | -0.301 | 143 | -21.92 | 2278 |
| ATMIN | -0.325 | 159 | -19.18 | 2388 |
| MTAP | -0.347 | 171 | -25.02 | 5227 |
| NPVF | -0.318 | 159 | -16.17 | 552 |
| STRN | -0.305 | 159 | -24.13 | 11758 |
| ASPHD2 | -0.335 | 157 | -20.49 | 1809 |
| KATNAL1 | -0.301 | 170 | -24.65 | 5910 |
| OR6P1 | -0.313 | 162 | -21.24 | 2045 |
| SRPX2 | -0.34 | 163 | -20.41 | 4806 |
| TMEM181 | -0.37 | 190 | -29.53 | 3547 |
| BMPR2 | -0.331 | 145 | -18.39 | 7805 |
| NYAP1 | -0.384 | 150 | -16.17 | 898 |
| CATG00000039203.1 | -0.358 | 161 | -17.46 | 1000 |
| CATG00000068853.1 | -0.31 | 158 | -19.65 | 1000 |
| NR4A3 | -0.308 | 183 | -31.97 | 3024 |
| PRICKLE1 | -0.349 | 154 | -20.77 | 3052 |
| CATG00000061733.1 | -0.392 | 157 | -16.17 | 1000 |
| NUP50 | -0.303 | 145 | -19.99 | 3360 |
| SMIM10 | -0.309 | 159 | -24.23 | 1126 |
| ZNF397 | -0.313 | 176 | -26.38 | 4824 |
| ANKHD1 | -0.312 | 148 | -21.54 | 1742 |
| ALKBH6 | -0.308 | 157 | -20.18 | 179 |
| ENTHD1 | -0.332 | 154 | -17.23 | 635 |
| SLC20A1 | -0.334 | 165 | -20.02 | 802 |
| TAB3 | -0.318 | 154 | -26.7 | 3985 |
| ARVCF | -0.347 | 152 | -19.62 | 893 |
| ZNF682 | -0.616 | 332 | -44.11 | 1587 |
| SENP5 | -0.39 | 162 | -20.49 | 4548 |
| PASK | -0.336 | 173 | -21.34 | 269 |
| GP1BA | -0.307 | 165 | -26.47 | 467 |
| CATG00000112890.1 | -0.346 | 165 | -15.78 | 1000 |
| HARBI1 | -0.338 | 165 | -18.55 | 2268 |
| LDB2 | -0.344 | 140 | -19.62 | 1102 |
| AIF1 | -0.36 | 151 | -19.09 | 121 |
| PRDM4 | -0.363 | 171 | -22.82 | 1344 |
| GALNT15 | -0.313 | 183 | -26.91 | 3841 |
| TCP11L2 | -0.319 | 183 | -28.69 | 2817 |
| ATP2B1 | -0.344 | 151 | -17.42 | 2912 |
| IFI44 | -0.361 | 168 | -22.49 | 267 |
| C9orf40 | -0.334 | 156 | -19.05 | 1467 |
| SPAAR | -0.322 | 157 | -18.29 | 995 |
| LRP6 | -0.34 | 150 | -16.37 | 5101 |
| CATG00000092952.1 | -0.302 | 167 | -21.77 | 1000 |
| CATG00000063513.1 | -0.345 | 156 | -21.4 | 1000 |
| GMCL1 | -0.342 | 165 | -23.23 | 2392 |
| TMED4 | -0.379 | 178 | -24.49 | 4410 |
| ANP32E | -0.353 | 175 | -25.89 | 2273 |
| PLEKHH1 | -0.316 | 155 | -16.13 | 2377 |
| C4orf45 | -0.364 | 145 | -18.83 | 569 |
| PROX2 | -0.361 | 168 | -27 | 2099 |
| PATE1 | -0.323 | 172 | -25.86 | 1134 |
| XYLT2 | -0.349 | 175 | -24.1 | 894 |
| IER5L | -0.312 | 172 | -17.78 | 1332 |
| DLG2 | -0.31 | 157 | -23.92 | 1899 |
| ANKRD63 | -0.325 | 142 | -19.67 | 3073 |
| ABHD5 | -0.313 | 170 | -17.9 | 4193 |
| SGTB | -0.66 | 309 | -36.97 | 4318 |
| CSMD3 | -0.311 | 149 | -20.68 | 1843 |
| DCDC1 | -0.389 | 162 | -19.03 | 1769 |
| FAM206A | -0.324 | 155 | -16.74 | 1930 |
| RFC5 | -0.311 | 141 | -20.58 | 514 |
| GPR19 | -0.311 | 161 | -19.34 | 310 |
| CDK2AP1 | -0.31 | 157 | -16.13 | 766 |
| CCSER1 | -0.308 | 144 | -20.32 | 3162 |
| IARS | -0.306 | 158 | -19.37 | 600 |
| KLHL14 | -0.344 | 159 | -24.52 | 4293 |
| FBP2 | -0.334 | 171 | -27.09 | 224 |
| ZNF677 | -0.324 | 156 | -17.63 | 1591 |
| FABP2 | -0.32 | 153 | -21.69 | 1791 |
| GPR158 | -0.333 | 153 | -18.51 | 2954 |
| ATRX | -0.317 | 164 | -20.48 | 3473 |
| IFRD2 | -0.328 | 148 | -16.17 | 459 |
| LDHC | -0.326 | 152 | -20.91 | 941 |
| NR3C1 | -0.365 | 154 | -25.41 | 727 |
| TMEM184B | -0.386 | 160 | -19.34 | 5074 |
| PPM1M | -0.393 | 159 | -18.09 | 785 |
| GATAD2B | -0.362 | 152 | -18.21 | 5452 |
| ZFP42 | -0.337 | 160 | -19.41 | 1310 |
| ANKRD28 | -0.366 | 158 | -19.32 | 3206 |
| ESD | -0.302 | 162 | -23.17 | 1097 |
| SLC9A4 | -0.332 | 143 | -20.13 | 1284 |
| NRBF2 | -0.354 | 160 | -26.2 | 883 |
| KIAA1024 | -0.306 | 157 | -19.31 | 3916 |
| SNAPC1 | -0.348 | 147 | -16.18 | 1445 |
| RAPH1 | -0.364 | 153 | -16.84 | 5755 |
| PRKG2 | -0.325 | 183 | -24.43 | 2338 |
| GABPB2 | -0.403 | 150 | -17.98 | 7286 |
| UBE2E1 | -0.31 | 150 | -16.57 | 1034 |
| MRRF | -0.306 | 150 | -17.38 | 8604 |
| PABPC1L2B | -0.339 | 146 | -21.32 | 1467 |
| ZFAND4 | -0.353 | 169 | -22.44 | 3080 |
| CELF6 | -0.342 | 151 | -19.75 | 466 |
| E2F3 | -0.386 | 145 | -22.08 | 3284 |
| SLC2A4 | -0.337 | 159 | -20.49 | 1628 |
| ISCA2 | -0.381 | 167 | -16.41 | 4759 |
| MDP1 | -0.342 | 157 | -16.18 | 109 |
| OR11H4 | -0.328 | 141 | -17.15 | 583 |
| CPEB2 | -0.304 | 152 | -23.32 | 3773 |
| GALNT2 | -0.352 | 164 | -22.04 | 4816 |
| DNAH12 | -0.35 | 148 | -16.17 | 496 |
| IGFBP7 | -0.368 | 158 | -16.87 | 558 |
| YARS2 | -0.334 | 144 | -18.13 | 2842 |
| NPPA | -0.349 | 169 | -23.18 | 300 |
| FAM151B | -0.324 | 142 | -22.14 | 5149 |
| CCDC40 | -0.344 | 141 | -16.33 | 838 |
| ZNF750 | -0.304 | 158 | -17.14 | 708 |
| LRP8 | -0.314 | 172 | -20.27 | 4649 |
| ATP10A | -0.343 | 156 | -15.78 | 2068 |
| TBL1XR1 | -0.329 | 153 | -16.17 | 6143 |
| TECTB | -0.662 | 308 | -36.61 | 1723 |
| NRBF2 | -0.372 | 160 | -26.2 | 813 |
| SYK | -0.338 | 148 | -28.34 | 2960 |
| FAM49B | -0.317 | 160 | -21.56 | 2549 |
| SLC9B1 | -0.376 | 166 | -19.09 | 189 |
| ALKBH6 | -0.308 | 157 | -20.18 | 456 |
| MYOCD | -0.338 | 145 | -16.17 | 5305 |
| CCL16 | -0.312 | 171 | -26.32 | 1067 |
| LURAP1L | -0.395 | 168 | -21.1 | 1699 |
| INTS8 | -0.405 | 161 | -18.67 | 1512 |
| NDFIP2 | -0.423 | 173 | -21.32 | 3557 |
| P2RY12 | -0.384 | 157 | -16.89 | 974 |
| EEF1AKMT1 | -0.315 | 148 | -16.42 | 349 |
| CELF5 | -0.313 | 168 | -24.1 | 359 |
| ABHD17C | -0.343 | 161 | -21.37 | 1251 |
| ADCY7 | -0.334 | 151 | -16.17 | 2630 |
| CLEC9A | -0.332 | 166 | -19.04 | 334 |
| ARPC3 | -0.359 | 155 | -17.15 | 721 |

**Table S4. Identification of tRF-1-S25 binding proteins via sequencing.**

| **Protein** | **Gene** | **NC** | **1-S25** |
| --- | --- | --- | --- |
| I4EP44 | HLA-C | 0 | 1334924 |
| A0A024RAM1 | POLR3G | 0 | 1011246 |
| Q8TAE8 | GADD45GIP1 | 0 | 688052.1 |
| B3KUZ8 |  | 0 | 678974 |
| A8K8K1 |  | 0 | 633090.6 |
| Q32Q75 | EIF4E | 0 | 398851.2 |
| A0A024R1Z6 | VAT1 | 0 | 351357.4 |
| Q86YS7 | C2CD5 | 0 | 340159.2 |
| Q9ULC4 | MCTS1 | 0 | 317273.9 |
| A0A024R0R4 | SAE1 | 0 | 298389.1 |
| A0A024R120 | TFCP2 | 0 | 281422.7 |
| Q15334 | LLGL1 | 0 | 262539.2 |
| B0AZV8 |  | 0 | 245740.8 |
| Q9UJX4 | ANAPC5 | 0 | 235978.9 |
| B0S8I6 | FAM50A | 0 | 214401.5 |
| J3KQ41 | COPS7B | 0 | 201657.9 |
| B4E1L0 | ADSS | 0 | 188841.3 |
| Q05397 | PTK2 | 0 | 176793.6 |
| Q8NAV1 | PRPF38A | 0 | 161016.8 |
| Q9BQ70 | TCF25 | 0 | 159227.3 |
| A0A7P0TBF9 | IFITM3 | 0 | 154702.4 |
| B7Z9C0 |  | 0 | 140285.9 |
| Q3B7A7 | GART | 0 | 135095.1 |
| Q9HC36 | MRM3 | 0 | 126974.6 |
| Q9H9J2 | MRPL44 | 0 | 124174.4 |
| Q9H9B1 | EHMT1 | 0 | 123226.1 |
| Q9NQ50 | MRPL40 | 0 | 122187.2 |
| F8WBH5 | PSME4 | 0 | 121784.3 |
| Q96GC5 | MRPL48 | 0 | 112201.1 |
| J3KSB0 | PPP4R1 | 0 | 92224.54 |
| M0QWZ7 | SARS2 | 0 | 83849.9 |
| Q9UBL3 | ASH2L | 0 | 76233.25 |
| Q9H6S0 | YTHDC2 | 0 | 61655.77 |
| A0A024R3V8 | TSNAX | 0 | 57940.96 |
| Q8NEF9 | SRFBP1 | 0 | 56500.71 |
| I3L387 | PLK1 | 0 | 45907.91 |
| A0A087X2D5 | MRPL45 | 0 | 41288.3 |
| Q8N1G2 | CMTR1 | 0 | 40055.32 |
| Q53HK8 |  | 0 | 19987.67 |
| Q53GZ2 |  | 0 | 18084.34 |
| Q4U2R6 | MRPL51 | 0 | 14729.82 |

**Table S5. Sequence-designed synthesized fragments.**

| **Name** | **Sequence** | **Supplier** |
| --- | --- | --- |
| **sgRNAs sequences** |  |  |
| sgNSun2-1 | GAACTCAAGATCGTGCCAGAGGG | Beijing Tsingke Biotech Co., Ltd |
| sgNSun2-2 | GTACCCAGTGATTCTCAGTGTGG | Beijing Tsingke Biotech Co., Ltd |
| **siRNA sequences** |  |  |
| NSun2 siRNA1 | CCAGAUAGAUGUGGACGGCAGGAAA | Beijing Tsingke Biotech Co., Ltd |
| NSun2 siRNA2 | CGGCUGGCACAGGAGGGAAUAUAUA | Beijing Tsingke Biotech Co., Ltd |
| DUSP1 siRNA1 | GGACATGCTGGATGCCTT | Beijing Tsingke Biotech Co., Ltd |
| DUSP1 siRNA2 | TAGCGTCAAGACATTTGCTGA | Beijing Tsingke Biotech Co., Ltd |
| **tRF-In sequences** |  |  |
| tRF-1-S25-In | AAACUGAGCACAGGACUUCCUUAGC | Beijing Tsingke Biotech Co., Ltd |
| tRF-5-V31-In | GCGAACGUGAUAACCACUACACUACAGAAGC | Beijing Tsingke Biotech Co., Ltd |
| **Identification of NSun2-KO** |  |  |
| Forward | 5'-AGCCTGAGGGCGAGGAAGAC-3' | Beijing Tsingke Biotech Co., Ltd |
| Reverse | 5'-GGGATCCAGTGCCCTCTTCTGAT-3 | Beijing Tsingke Biotech Co., Ltd |
| **Primers for qRT-PCR** |  |  |
| 1-S25 RT primer | 5'-GTCGTATCGACTGCAGGGTCCGAGG  TATTCGCAGTCGATACGACAAACTG-3' | Beijing Tsingke Biotech Co., Ltd |
| 5-V31 RT primer | 5'-GTCGTATCGACTGCAGGGTCCGAGG  TATTCGCAGTCGATACGAGCGAAC-3' | Beijing Tsingke Biotech Co., Ltd |
| 1-S25-Forward | 5'-GCGCTAAGGAAGTCCTGTGCT-3' | Beijing Tsingke Biotech Co., Ltd |
| 1-S25-Reverse | 5'-ACTGCAGGGTCCGAGGTATT-3' | Beijing Tsingke Biotech Co., Ltd |
| 5-V31-Forward | 5'-GCGCTTCTGTAGTGTAGTGGTTATCAC-3' | Beijing Tsingke Biotech Co., Ltd |
| 5-V31-Reverse | 5'-ACTGCAGGGTCCGAGGTATT-3' | Beijing Tsingke Biotech Co., Ltd |
| mouse-U6 Forward | 5'-CGCTTCGGCAGCACATATAC-3' | Beijing Tsingke Biotech Co., Ltd |
| mouse-U6-Reverse | 5'-CACGAATTTGCGTGTCATCC-3' | Beijing Tsingke Biotech Co., Ltd |
| mouse-Col1a-Forward | 5'-GCTCCTCTTAGGGGCCACT-3' | Beijing Tsingke Biotech Co., Ltd |
| mouse-Col1a-Reverse | 5'-CCACGTCTCACCATTGGGG-3' | Beijing Tsingke Biotech Co., Ltd |
| Mouse-NSun2-Forward | 5'-ACACTGAGAATCACTGGGTACA-3' | Beijing Tsingke Biotech Co., Ltd |
| mouse-NSun2-Reverse | 5'-CCAGCTTAGTGGTTGTGGAACT-3' | Beijing Tsingke Biotech Co., Ltd |
| mouse-CD34-Forward | 5'-ATCCCCATCAGTTCCTACCAAT-3' | Beijing Tsingke Biotech Co., Ltd |
| mouse-CD34-Reverse | 5'-TGGTGTGGTCTTACTGCTGTC-3' | Beijing Tsingke Biotech Co., Ltd |
| mouse-β-actin-Forward | 5'-GGCTGTATTCCCCTCCATCG-3' | Beijing Tsingke Biotech Co., Ltd |
| mouse-β-actin-Reverse | 5'-CCAGTTGGTAACAATGCCATGT-3' | Beijing Tsingke Biotech Co., Ltd |
| mouse-c-met-Forward | 5'-GTGAACATGAAGTATCAGCTCCC-3' | Beijing Tsingke Biotech Co., Ltd |
| mouse-c-met-Reverse | 5'-TGTAGTTTGTGGCTCCGAGAT-3' | Beijing Tsingke Biotech Co., Ltd |
| mouse-Vegfr2-Forward | 5'-TTTGGCAAATACAACCCTTCAGA-3' | Beijing Tsingke Biotech Co., Ltd |
| Mouse-Vegfr2- Reverse | 5'-GCAGAAGATACTGTCACCACC-3' | Beijing Tsingke Biotech Co., Ltd |
| human-U6-Forward | 5'-TGCTTCGGCAGCACATATAC-3' | Beijing Tsingke Biotech Co., Ltd |
| human-U6-Reverse | 5'-TCACGAATTTGCGTGTCATC-3' | Beijing Tsingke Biotech Co., Ltd |
| human-FAK-Forward | 5'-AGTGGACCAGGAAATTGCTTTG-3' | Beijing Tsingke Biotech Co., Ltd |
| human-FAK-Reverse | 5'-GTGTTTTGGCCTTGACAGAATC-3' | Beijing Tsingke Biotech Co., Ltd |
| human-DUSP1-Forward | 5'-GCCTTGCTTACCTTATGAGGAC-3' | Beijing Tsingke Biotech Co., Ltd |
| human-DUSP1-Reverse | 5'-GGGAGAGATGATGCTTCGCC-3' | Beijing Tsingke Biotech Co., Ltd |
| human-NSun2-Forward | 5'-GAACTTGCCTGGCACACAAAT-3' | Beijing Tsingke Biotech Co., Ltd |
| human-NSun2-Reverse | 5'-TGCTAACAGCTTCTTGACGACTA-3' | Beijing Tsingke Biotech Co., Ltd |
| human-GAPDH-Forward | 5'-GGAGCGAGATCCCTCCAAAAT-3' | Beijing Tsingke Biotech Co., Ltd |
| human-GAPDH-Reverse | 5'-GGCTGTTGTCATACTTCTCATGG-3' | Beijing Tsingke Biotech Co., Ltd |

**Table S6. Antibodies for analysis.**

| **Antibody** | **Source** | **Cat No.** |
| --- | --- | --- |
| CD31 | Abcam | ab28364 |
| CD34 | Solarbio | K009404P |
| α-SMA | Abcam | ab5694 |
| VE-cad | R&D system | AF1002 |
| NSun2 | Abcam | 128243 |
| FAK | Proteintech | 66258-1-lg |
| p-FAK(Tyr576) | Affinity | AF3397 |
| DUSP1 | Affinity | AF5286 |
| GAPDH | Abcam | ab8245 |
